# The non-receptor tyrosine kinase Pyk2 modulates acute locomotor effects of cocaine in D1 receptor-expressing neurons of the nucleus accumbens

**DOI:** 10.1038/s41598-020-63426-5

**Published:** 2020-04-20

**Authors:** Benoit de Pins, Enrica Montalban, Peter Vanhoutte, Albert Giralt, Jean-Antoine Girault

**Affiliations:** 1 Inserm UMR-S 1270, Paris, 75005 France; 2 Sorbonne Université, Faculty of Sciences and Engineering, Paris, 75005 France; 3 Institut du Fer à Moulin, Paris, 75005 France; 4 Inserm UMR-S 1130, Neurosciences Paris Seine, Paris, 75005 France; 5 CNRS UMR 8246, Paris, 75005 France; 6 Present Address: Department of Plant and Environmental Sciences, Weizmann Institute of Science, Rehovot, 7610001 Israel; 7 Present Address: BFA - Unité de Biologie Fonctionnelle et Adaptative - CNRS UMR 8251, Paris University, Paris, 75205 France; 8 Present Address: Departament de Biomedicina, Facultat de Medicina, Institut de Neurociències, Universitat de Barcelona, Institut d’Investigacions Biomèdiques August Pi i Sunyer (IDIBAPS), Barcelona 08036, Spain and Centro de Investigación Biomédica en Red sobre Enfermedades Neurodegenerativas (CIBERNED), Madrid, 28031 Spain

**Keywords:** Molecular neuroscience, Reward, Neuroscience

## Abstract

The striatum is critical for cocaine-induced locomotor responses. Although the role of D1 receptor-expressing neurons is established, underlying molecular pathways are not fully understood. We studied the role of Pyk2, a non-receptor, calcium-dependent protein-tyrosine kinase. The locomotor coordination and basal activity of Pyk2 knock-out mice were not altered and major striatal protein markers were normal. Cocaine injection increased Pyk2 tyrosine phosphorylation in mouse striatum. Pyk2-deficient mice displayed decreased locomotor response to acute cocaine injection. In contrast, locomotor sensitization and conditioned place preference were normal. Cocaine-activated ERK phosphorylation, a signaling pathway essential for these late responses, was unaltered. Conditional deletion of Pyk2 in the nucleus accumbens or in D1 neurons reproduced decreased locomotor response to cocaine, whereas deletion of Pyk2 in the dorsal striatum or in A_2A_ receptor-expressing neurons did not. In mice lacking Pyk2 in D1-neurons locomotor response to D1 agonist SKF-81297, but not to an anticholinergic drug, was blunted. Our results identify Pyk2 as a regulator of acute locomotor responses to psychostimulants. They highlight the role of tyrosine phosphorylation pathways in striatal neurons and suggest that changes in Pyk2 expression or activation may alter specific responses to drugs of abuse, or possibly other behavioral responses linked to dopamine action.

## Introduction

The striatum is the main input structure of the basal ganglia, involved in motor coordination, motivation, and action selection. The GABAergic striatal projection neurons (SPNs, a.k.a. medium-size spiny neurons) constitute the vast majority of striatal neurons (~95% in rodents). They integrate cortical and thalamic excitatory inputs and are modulated by dopaminergic inputs and striatal interneurons^1^. Two major populations of SPNs can be distinguished according to their projection targets and their molecular profile^1–3^. SPNs enriched in enkephalin, D2 dopamine (D2R), and A_2A_ adenosine (A_2A_R) receptors project to the external globus pallidus (GPe) thus participating in the indirect striatonigral pathway, while SPNs enriched in substance P, dynorphin, and D1 dopamine receptor (D1R) innervate the internal globus pallidus (GPi) and the substantia nigra pars reticulata (SNr), forming the direct pathway^1,2,4^. Striatal dysfunction is associated with several pathologies ranging from movement disorders in Parkinson’s or Huntington’s diseases to addiction^5^. The striatum is a major site of action of drugs of abuse, which all share the ability to increase extracellular dopamine levels in the ventral part of the striatum, the nucleus accumbens ^6^. The signaling pathways involved in the action of glutamate, dopamine, and other neurotransmitters in SPNs have been extensively studied^7^. Although attention mostly focused on pathways involving protein serine/threonine phosphorylation, tyrosine phosphorylation is also likely to be important^8^. The regulation and functional importance of tyrosine kinases in striatal neurons are however poorly characterized.

Pyk2 is a Ca^2+^-dependent non-receptor tyrosine kinase highly expressed in forebrain neurons^9^ where it is activated by neuronal activity and excitatory neurotransmission^10–12^. Pyk2 can associate with the NMDA receptor complex and has a role in synaptic plasticity^11,13–15^. Ca^2+^ triggers Pyk2 autophosphorylation on Tyr-402, which recruits and activates Src-family kinases (SFKs)^16,17^. In turn, SFKs phosphorylate other residues in Pyk2 and associated proteins, and initiate multiple signaling pathways. The striatal-enriched protein tyrosine phosphatase (STEP) dephosphorylates Pyk2^18^. Activated Pyk2 regulates many cellular functions^19,20^ and Pyk2 is associated with several pathologies including cancer^21^, inflammatory diseases^20^, Huntington’s^15^, Alzheimer’s diseases^22–25^, and chronic stress sequelae^26^. Work with Pyk2 knockout mice showed that Pyk2 in the hippocampus is involved in spatial memory and regulates spine density and morphology, as well as long-term potentiation^15^ and depression^26^. These studies illustrate the role of Pyk2 in synaptic functions and behavior in physiological and pathological conditions.

Here, we explored whether Pyk2 deficit also altered striatal function, using constitutive and conditional KO mice. We found that Pyk2 protein was relatively enriched in D1R-expressing SPNs in the ventral striatum. Pyk2 phosphorylation was increased following cocaine injection. Deletion of Pyk2 reduced the acute locomotor response to cocaine but, surprisingly, neither locomotor sensitization nor conditioned place preference. Use of conditional deletion showed a specific involvement of Pyk2 in D1R-expressing SPNs of the nucleus accumbens (NAc) in cocaine-induced locomotor response.

## Results

### Pyk2 protein is enriched in ventral D1 SPNs

**Figure 1:**
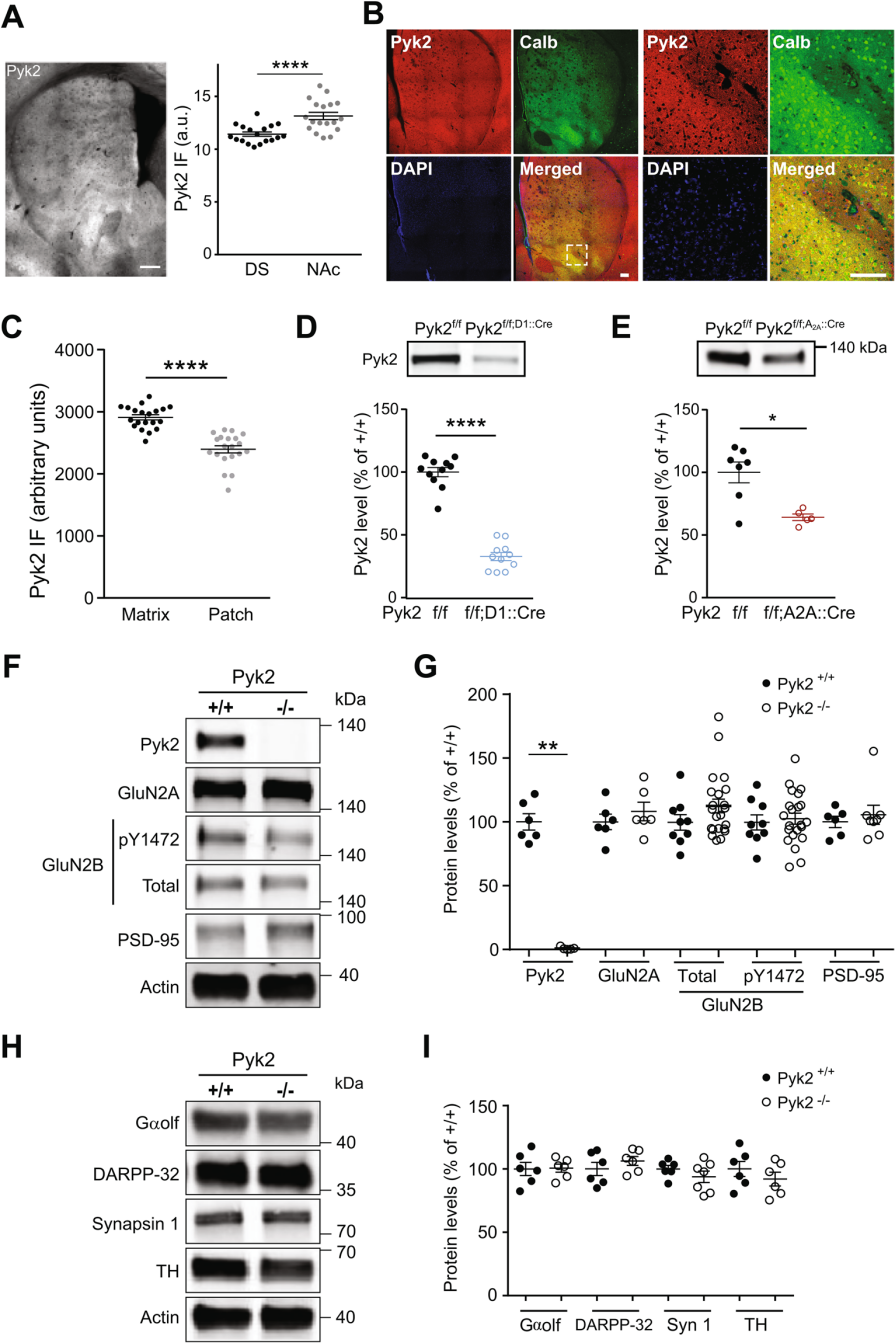
Expression of Pyk2 in the striatum and effects of its deletion on protein markers. (**A**), Characterization of Pyk2 expression in the striatum. Left panel, distribution of Pyk2 striatal immunoreactivity in a coronal brain section of a wild type mouse. Right panel, Pyk2 labelling intensity in the dorsal striatum (DS) and nucleus accumbens (NAc) measured in 6 sections per mouse in 3 mice. Scale bar: 300 µm. (**B**), Distribution of Pyk2 (red) and calbindin (green) immunoreactivity, and DAPI (blue) in the striatum of wild-type mice. The right panel shows higher magnification of the area indicated by a white dashed square in the left panel. Scale bars: 150 μm. (**C**), Pyk2 labelling intensity in striatal matrix as defined as calbindin-enriched areas (n = 20 regions from 3 mice). (**D**), Immunoblot of Pyk2 protein in the striatum of Pyk2^f/f;D1::Cre^ and matched Pyk2^f/f^ mice (n = 11 mice per group). (**E**), Immunoblot of Pyk2 protein in the striatum of Pyk2^f/f;A2A::Cre^ (n = 5 mice) and matched Pyk2^f/f^ mice (n = 7). (**F**), Immunoblot of Pyk2, NMDA receptors subunits (total and pTyr1472-GluN2B, GluN2A), PSD-95 and actin as loading control, in 4-month Pyk2^+/+^ and Pyk2^−/−^ mice. (**G**), Densitometry quantification of results as in F. Data were normalized to actin for each sample and expressed as percentage of wild type mean density (6–22 mice per group). (**H**), Immunoblot of Gαolf, DARPP-32, synapsin 1, tyrosine hydroxylase (TH), and actin. (**I**), Results as in H were quantified and analyzed as indicated in G (6–7 mice per group). In A, C-E, G, and I, means ± SEM are indicated, 2-tailed Mann and Whitney test, *p < 0.05, **p < 0.01, ****p < 0.0001. See Supplementary Table 1 for all details of statistical analyses and Supplementary Figures 2 and 3 for full length blots.

We used immunohistochemistry to evaluate the regional distribution of Pyk2 protein in the striatum. Pyk2 immunoreactivity appeared slightly stronger in the NAc than in the DS ( + 15%, Fig. 1A right panel, unpaired t test, t_34_ = 4.3, p = 10^−4^, see Supplementary Table 1 for details of all statistical analyses), in agreement with a previous study of mRNA distribution^27^. Since Pyk2 striatal immunoreactivity displayed an irregular pattern, we assessed whether its distribution followed the neurochemically-defined patch/matrix compartmentalization^28–30^ defined by calbindin, a matrix-enriched protein (Fig. 1B). Pyk2 appeared more expressed in calbindin-positive neurons indicating an enrichment of Pyk2 in matrix compartment (+21%, Fig. 1C, n = 20 regions from 3 mice, two-tailed Mann Whitney’s test, U_20,20_ = 12, p < 10^−4^). To evaluate the relative expression of Pyk2 in neurons of the direct and indirect pathways, we then compared its decrease following conditional deletion in either of these populations, as compared to their matched controls. Pyk2 striatal protein levels were decreased by 67% in Pyk2^f/f;D1::Cre^ mice (Fig. 1D, n = 11 mice per group, Mann-Whitney test, U_11,11_ = 0, p < 10^−4^). This result should be taken with caution since the expression of D1R is wider during development of the striatum than in the adult^31,32^ and could lead to a broader developmental action of D1::Cre and subsequent overestimation of the apparent enrichment of Pyk2 in D1 neurons. However, Pyk2 decrease in Pyk2^f/f;A2A::Cre^ mice was limited to 36%, as compared to matched Pyk2^f/f^ mice (Fig. 1E, Mann-Whitney test, U_7,5_ = 4, p = 0.03), supporting the hypothesis of a relatively higher expression of Pyk2 protein in D1R-expressing SPNs than in A_2A_R-positive neurons. In conclusion, although Pyk2 is expressed in all SPNs it appears to be slightly more abundant in D1 neurons, and in the DS matrix and the NAc.

### Pyk2 deletion does not alter striatal proteins

In the hippocampus of Pyk2^-/-^ mice, we previously observed decreased levels of several synaptic proteins^15^. In the striatum of Pyk2^-/-^ mice (Fig. 1F,G), Pyk2 was not detectable (Mann-Whitney test, U_6,6_ = 0, p = 0.002) but neither GluN2A nor PSD-95 levels were altered (Mann-Whitney test, GluN2A, U_6,6_ = 13, p = 0.48; PSD-95, U_6,8_ = 24, p > 0.99). GluN2B levels and its form phosphorylated at Tyr1472 were also unchanged in the striatum of Pyk2^-/-^ mice (GluN2B, U_9,22_ = 68, p = 0.19; pY1472-GluN2B, U_9,22_ = 93, p = 0.81). To examine the status of the striatum, we measured the SPN-enriched proteins Gαolf^33^ and dopamine- and cAMP-regulated neuronal phosphoprotein (DARPP-32)^34,35^ (Fig. 1H,I). There was no modification in the levels of Gαolf (U_6,6_ = 17, p = 0.90) and DARPP-32 (U_6,6_ = 11, p = 0.31, Fig. 1I). A general presynaptic marker, synapsin 1 (U_6,7_ = 15, p = 0.45) and a dopamine terminals marker, tyrosine hydroxylase (U_6,6_ = 12, p = 0.39, Fig. 1H,I) were also unchanged. These results show that the absence of Pyk2 did not alter synaptic or neuronal markers in the striatum.

### Pyk2 knockout does not impair motor coordination

**Figure 2:**
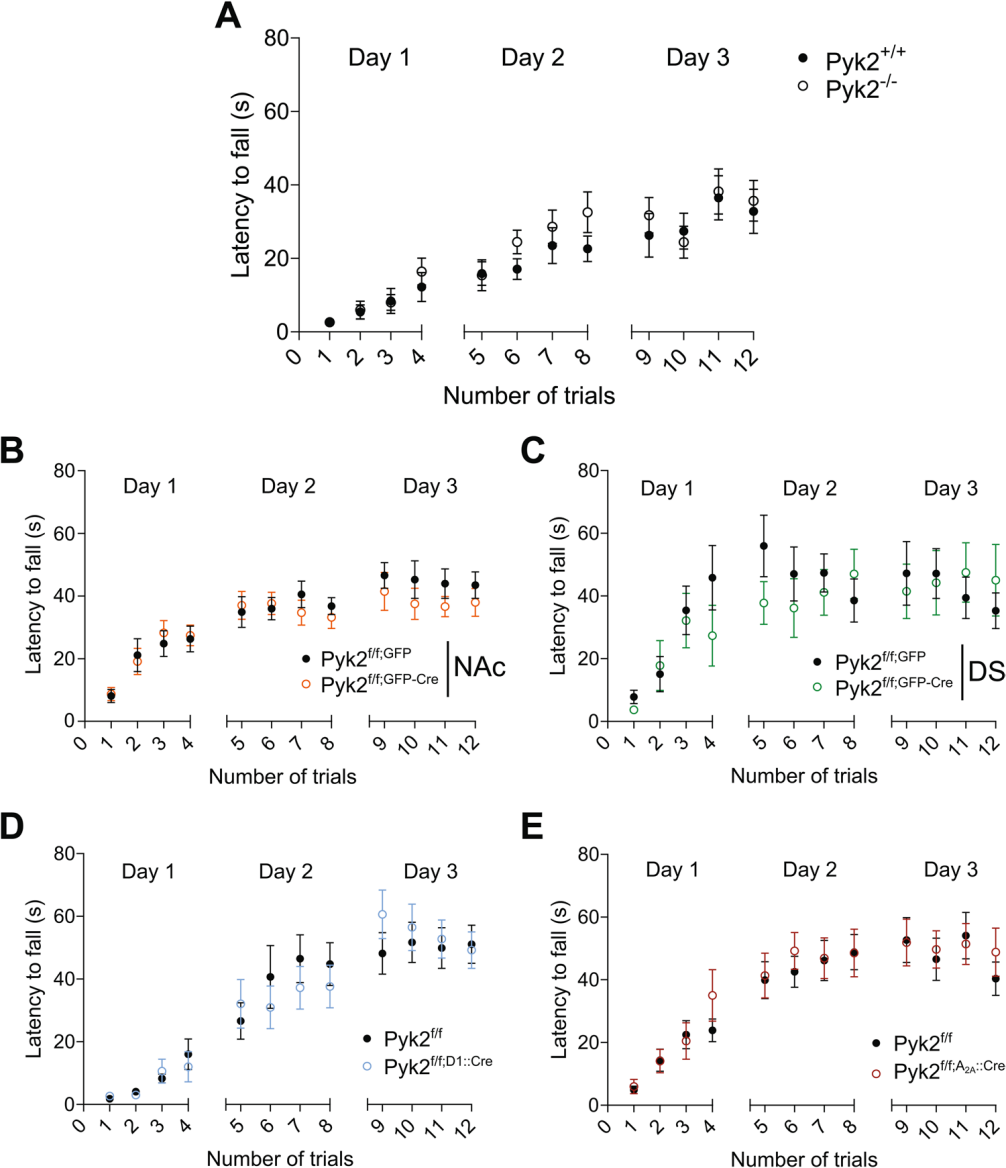
Motor skill learning is not altered in Pyk2 mutant mice. Various Pyk2 mutant mice and their matched controls were trained on a rotarod at accelerating speed (4–40 rpm in 5 min), with four sessions per day for three consecutive days and the latency to fall was recorded for each trial. (**A**), Pyk2^+/+^ and Pyk2^-/-^ mice, 9 per genotype. (**B**), Mice injected in the NAc with AAV expressing GFP alone or GFP and Cre (Pyk2^f/f;NAc,GFP^ and Pyk2^f/f;NAcS,GPF-Cre^ mice, 17 and 14 per group, respectively). (**C**), Mice injected with the same AAVs in the DS (Pyk2^f/f;DS,GFP^ and Pyk2^f/f;DS,GPF-Cre^ mice, 8 and 7 per group, respectively). (**D**), Pyk2^f/f^ and Pyk2^f/f;D1::Cre^ conditional KO mice, 14 and 9 mice per genotype, respectively. (**E**), Pyk2^f/f^ and Pyk2^f/f;A2A::Cre^ conditional KO mice, 14 and 12 mice per genotype, respectively. See Supplementary Table 1 for detailed statistical analyses.

To study the role of Pyk2 in the striatum, we first assessed motor coordination. Pyk2^+/+^ and Pyk2^-/-^ mice were trained on an accelerating rotarod for 3 days and their motor coordination was evaluated by measuring the time they were able to stay on the rod during each trial. Mice of both genotypes displayed similar performances, improving their ability to stay on the rod across trials (Fig. 2A, two-way ANOVA, trial number effect, F_11,191_ = 14.03, p < 10^−4^; genotype effect, F_1,191_ = 2.57, p = 0.11, see Supplementary Table 1 for details of all statistical analyses). Similarly, there was no alteration of rotarod performance following specific AAV-mediated Cre deletion of Pyk2 in the NAc (Fig. 2B, two-way ANOVA, trial number effect, F_11,345_ = 12.20, p < 10^−4^; genotype effect: F_1,345_ = 1.83, p = 0.18) or in the DS (Fig. 2C, two-way ANOVA, trial number effect, F_11,155_ = 4.99, p < 10^−4^; genotype effect: F_1,155_ = 1.07, p = 0.30). Conditional deletion of Pyk2 did not impair rotarod performance in Pyk2^f/f;D1::Cre^ mice (Fig. 2D, two-way ANOVA, trial number effect, F_11,298_ = 22.46, p < 10^−4^; genotype effect: F_1,298_ = 0.02, p = 0.88) or in Pyk2^f/f;A2A::Cre^ mice (Fig. 2E, two-way ANOVA, trial number effect, F_11,286_ = 14.47, p < 10^−4^; genotype effect: F_1,286_ = 0.84, p = 0.36). These results showed that the absence of Pyk2 did not alter motor coordination in the rotarod test.

### Pyk2 is activated by cocaine and involved in its acute locomotor effects but not in their sensitization

**Figure 3:**
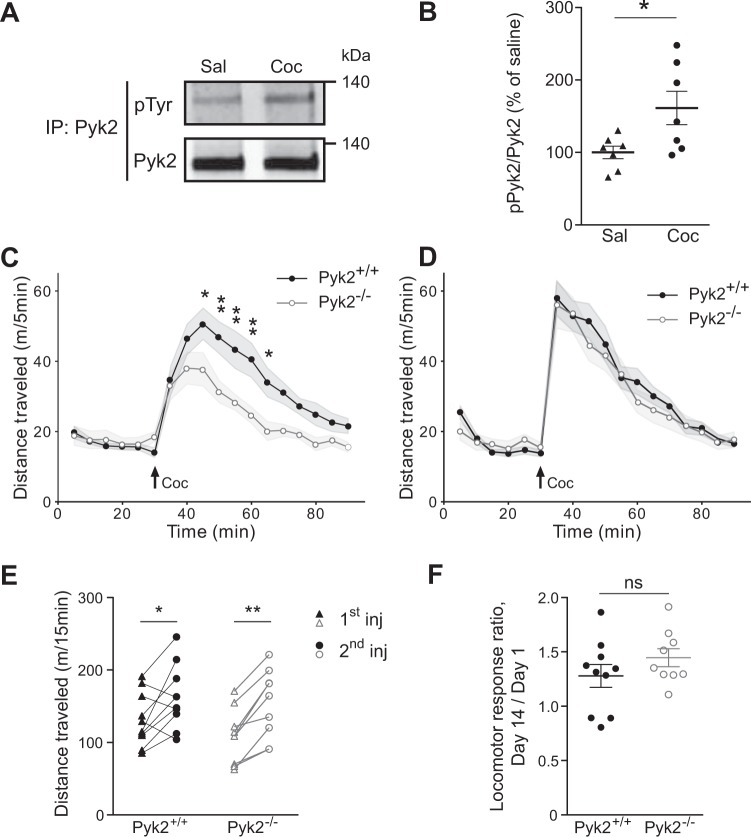
Pyk2 is implicated in acute cocaine responses. (**A**), Immunoblot for phospho-tyrosine and total Pyk2 after Pyk2 immunoprecipitation (IP) from striatal extracts of wild-type mice killed 10 min after i.p. injection of saline (Sal) or cocaine (20 mg/kg, Coc). (**B**), Densitometry quantification of results as in A. Data are ratios of phospho/total Pyk2 for each sample and expressed as percentage of the mean ratios in saline-treated samples (n = 7 mice per group). (**C**), Locomotor activity of Pyk2^+/+^ (n = 10 mice) and Pyk2^-/-^ mice (n = 9) after a first cocaine injection (20 mg/kg, Coc, arrow) 30 min after mice were placed in the open field. (**D**), The same mice received a second injection of cocaine in the same conditions as in C, 13 days later. (**E**), Pairwise comparison of the distance traveled 0–15 minutes following the first and the second injection in Pyk2^+/+^ and Pyk2^−/−^ mice (from data in C and D). (**F**), Sensitization ratios calculated as the distance traveled 0–15 min after the second injection (day 14) divided by the distance traveled during the same period after the first (day 1) cocaine injection (from data in E), in Pyk2^+/+^ and Pyk2^−/−^ mice. (**B-F**), Data are means ± SEM, indicated by a shaded area in C and D, and horizontal bars in B, E and F. t-test in B and F, 2-way ANOVA followed by post-hoc Sidak’s multiple comparisons test in C-E. ns, not significant, *p < 0.05, **p < 0.01. See Supplementary Table 1 for detailed statistical analyses and Supplementary Figure 3 for full length blots.

Considering the relative enrichment of Pyk2 in the NAc, a region involved in reward response and addiction, we assessed a possible role of Pyk2 in the effects of cocaine. We immunoprecipitated Pyk2 from striatal extracts of mice killed 10 min after the injection of saline vehicle or cocaine (20 mg/kg), a time previously shown to allow detection of cocaine-induced increased tyrosine phosphorylation of SFK or NMDA-R^8^, and measured its tyrosine phosphorylation by immunoblotting (Fig. 3A). Tyrosine phosphorylation of Pyk2 was increased 10 min after cocaine injection, indicating its activation in response to cocaine injection (Fig. 3B, unpaired t test, t_12_ = 2.49, p = 0.029). We then explored a possible role of Pyk2 in the acute response to cocaine by comparing locomotor activity of Pyk2^+/+^ and Pyk2^-/-^ mice after cocaine injection. Basal locomotor activity was similar in the two groups of mice, whereas cocaine-induced hyperlocomotion was decreased in Pyk2^-/-^ mice (Fig. 3C, two-way ANOVA, genotype effect, F_1,306_ = 39.05, p < 10^−4^, see Supplementary Table 1 for details of all statistical analyses). Repeated cocaine exposure increases behavioral responses due to a sensitization mechanism^36^, which is clearly visible following a single cocaine administration^37^. We therefore injected cocaine a second time, thirteen days later, to assess locomotor sensitization (Fig. 3D). Locomotor response to the second injection of cocaine was increased in both Pyk2^+/+^ and Pyk2^-/-^ mice but there was no difference between the two genotypes (Fig. 3D, two-way ANOVA, genotype effect, F_1,306_ = 1.59, p = 0.21). Paired analysis showed a significant increase in locomotor activity in both genotypes (Fig. 3E, two-way ANOVA, injection effect: F_1,17_ = 25.49, p < 10^−4^; Sidak’s multiple comparisons post hoc tests: Pyk2^+/+^, t_17_ = 2.98, p = 0.017, Pyk2^-/-^, t_17_ = 4.13, p = 0.0014). To evaluate the degree of sensitization we compared the sensitization ratio (total locomotion during the first 15 min after the 1^st^ injection/total locomotion in the same period after the second injection) in the 2 genotypes. These ratios were similar in the two groups (Fig. 3F, unpaired t test, t_17_ = 1.24, p = 0.23). To test the possibility that decreased locomotor activity was linked to increased time spent in stereotypies we quantified the number of rearings and the time spent grooming during 40 min after the first cocaine injection, in 6 randomly selected mice in each group (Supplementary Fig. 1). There was no significant difference between Pyk2^+/+^ and Pyk2^-/-^ mice for these two parameters. The limited number of rearings (<100) and time spent grooming (<100 sec) during the 40-min after cocaine injection make it unlikely that these activities interfered with locomotor activity. Therefore our results indicate a predominant role of Pyk2 in the acute cocaine-induced locomotor response but not in its sensitization.

### Pyk2 deletion in NAc, but not DS, recapitulates the effects of full KO on cocaine-induced locomotion

**Figure 4:**
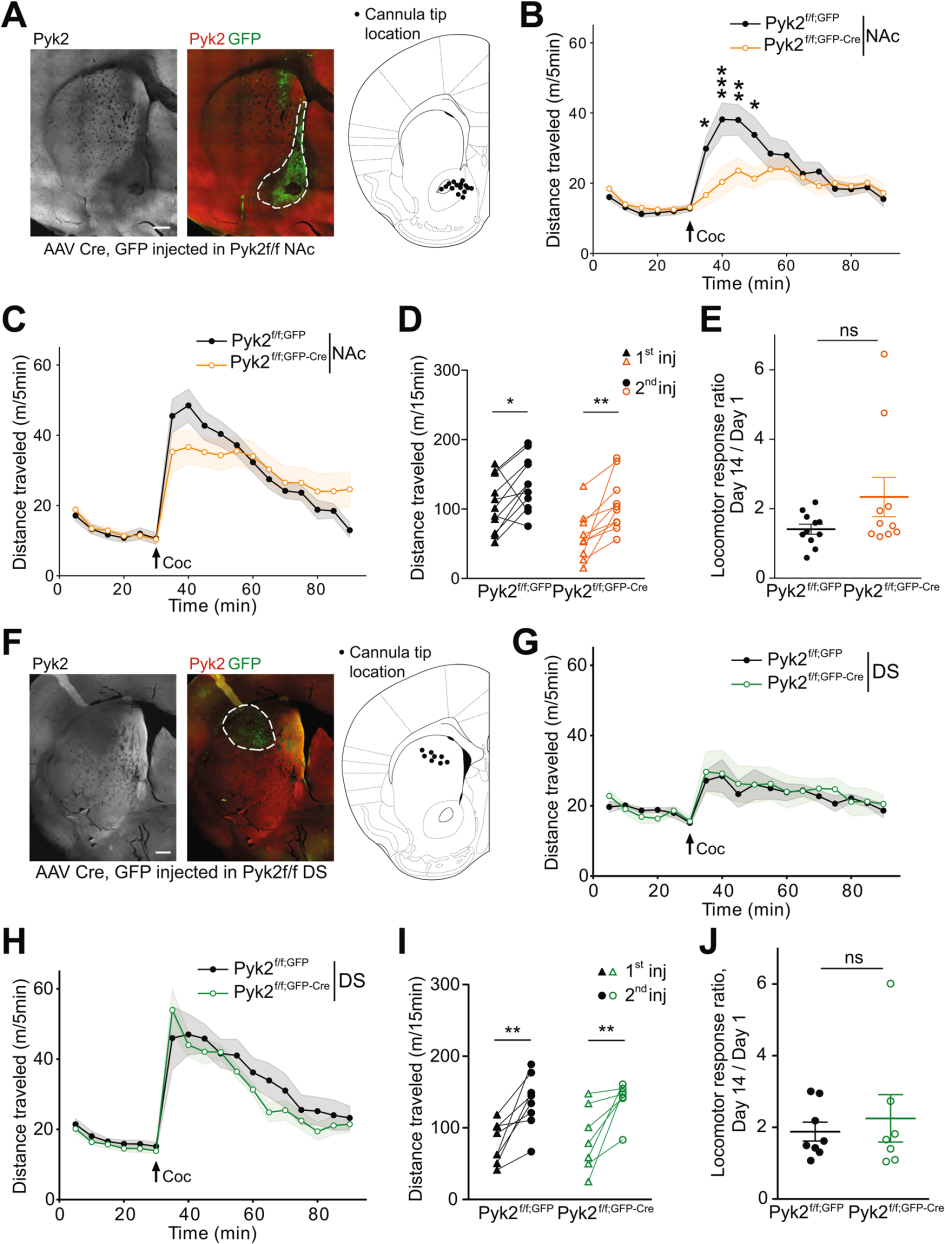
Specific deletion of Pyk2 in the NAc but not in the DS alters the acute locomotor response to cocaine. (**A**), Pyk2^f/f^ mice were bilaterally injected in the NAc with AAV expressing GFP (Pyk2^f/f;NAc,GFP^) or Cre and GFP (Pyk2^f/f;NAc,GFP-Cre^). A representative section through a Pyk2^f/f;NAc,GFP-Cre^ striatum is shown (left panel, Pyk2 immunofluorescence, right panel, Pyk2, red, and GFP, green, double fluorescence). The location of the tips of injecting cannula in mice used for further analysis is summarized (right panel, schematic brain section from Paxinos and Franklin^64^). (**B**), Locomotor activity of the mice injected in the NAc after the first injection of cocaine (20 mg/kg, Coc, arrow), as in Fig. 3C (Pyk2^f/f;NAc,GFP^, n = 11 mice) or Pyk2^f/f;NAc,GFP-Cre^, n = 10 mice). (**C**), The same mice received a 2^nd^ injection of cocaine, 13 days later. (**D**), Pairwise comparison of the distance traveled 0–15 minutes following the first and the second injection in Pyk2^f/f;NAc,GFP^ and Pyk2^f/f;NAc,GFP-Cre^ mice (from data in B and C). (**E**), Sensitization ratio as in Fig. 3F (distance traveled 0–15 min after the second injection [day 14, D] / distance traveled during the same period after the first injection [day 1, C]). (**F-J**), Same as in A-E, except that mice were injected into the DS, not the NAc. (**F**), Anatomical verification as in A. (**G**, **H**), Locomotor activity of Pyk2^f/f;DS,GFP^ (n = 8 mice) and Pyk2^f/f;DS,GFP-Cre^ mice (n = 7) after the first (**G**) and the second (**H**, 13 days later) injection of 20 mg/kg cocaine. **I**, Pairwise comparison of the distance traveled 0–15 minutes following the first and the second injection in Pyk2^f/f;DS,GFP^ and Pyk2^f/f;DS,GFP-Cre^ mice (from data in G and H). **J**, Sensitization ratios, as in E, in Pyk2^f/f;DS,GFP^ and Pyk2^f/f;DS,GFP-Cre^ mice. (**B-E** and **G-J**), Data are means ± SEM, indicated by a shaded area in B, C, G, and H, and horizontal bars in E and J. In B-D and G-I, data were analyzed with 2-way ANOVA followed by post-hoc Sidak’s multiple comparisons test, and in E and I by Mann and Whitney test. ns, not significant, *p < 0.05, **p < 0.01, ***p  <  0.001. See Supplementary Table 1 for detailed statistical analyses.

To identify the striatal region implicated in the effects of Pyk2 deletion, we studied cocaine responses in Pyk2^f/f^ mice bilaterally injected with AAV-GFP-Cre in the NAc. The behavior was investigated 3 weeks after the injection and at the end of the behavioral tests, the mice were sacrificed and the position of the tip of the injection needle, Pyk2 immunoreactivity, and GFP expression were checked (Fig. 4A). In mice injected with AAV-GFP-Cre, Pyk2 immunoreactivity was decreased in the GFP-expressing area (Fig. 4A). Animals in which the injection was not correctly located were not included in the statistical analysis. In mice injected with AAV-GFP-Cre in the NAc, basal locomotor activity was unchanged, but acute locomotor response to cocaine was decreased as compared to mice injected with AAV-GFP (Fig. 4B, two-way ANOVA, Cre effect, F_1,342_ = 12.94, p = 0.0004, see Supplementary Table 1 for details of all statistical analyses). Following a second injection of cocaine at day 14, the locomotor response increased in the two groups of mice and the difference between them was blunted (Fig. 4C, Cre effect, F_1,342_ = 0.015, p = 0.90). Paired analysis showed sensitization in both AAV-GFP and AAV-GFP-Cre mice (Fig. 4D, two-way ANOVA, injection effect: F_1,19_ = 20.05, p = 0.0003; Sidak’s multiple comparisons post hoc tests: Pyk2^f/f^, AAV-GFP, t_19_ = 2.58, p = 0.036, Pyk2^f/f^, AAV-GFP-Cre, t_19_ = 3.73, p = 0.003). The response ratio was not significantly different between the two groups (Fig. 4E, unpaired Mann-Whitney test, U_11,10_ = 39, p = 0.28). Bilateral injection of AAV-GFP or AAV-GFP-Cre in the DS (Fig. 4F) did not alter the locomotor effects of the first cocaine injection (Fig. 4G, Cre effect, F_1,234_ = 0.55, p = 0.46). After the second injection of cocaine locomotor activity was slightly decreased in AAV-GFP-Cre-injected mice as compared to AAV-GFP-injected animals (Fig. 4H, Cre effect, F_1,234_ = 7.93, p = 0.0053). Given the small amplitude of the difference its biological significance is uncertain. Both AAV-GFP and AAV-GFP-Cre mice were sensitized (Fig. 4I, two-way ANOVA, injection effect: F_1,13_ = 26.55, p = 0.0002; Sidak’s multiple comparisons post hoc tests: Pyk2^f/f^, AAV-GFP, t_13_ = 3.86, p = 0.04, Pyk2^f/f^, AAV-GFP-Cre, t_13_ = 3.44, p = 0.009). The sensitization ratios were similar in the two groups of animals (Fig. 4J, unpaired Mann-Whitney test, U_14,12_ = 27, p = 0.92). These results provide evidence that the NAc is the region implicated in the consequences of Pyk2 deficit in the impaired locomotor response to the first cocaine injection.

### Pyk2 conditional deletion in cells expressing D1R, but not A_2A_R receptor impairs acute cocaine-induced locomotion

**Figure 5:**
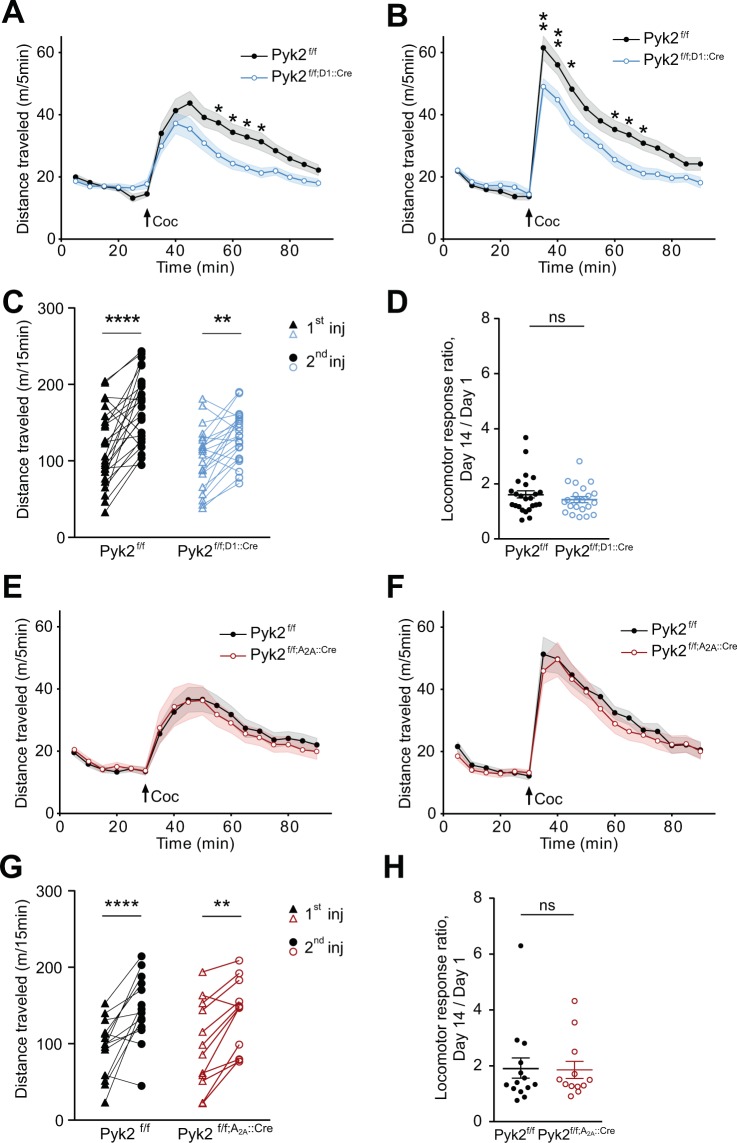
Specific deletion of Pyk2 in cells expressing D1 but not in A_2A_ receptor alters the locomotor response to cocaine. A-B, Locomotor activity of Pyk2^f/f^ (n = 24 mice) and Pyk2^f/f;D1::Cre^ mice (n = 22) after the first (**A**) and the second (**B**, 13 days later) injection of cocaine (20 mg/kg, Coc, arrow) as in Fig. 3C,D. (**C**), Pairwise comparison of the distance traveled 0–15 minutes following the first and the second injection in Pyk2^f/f^ and Pyk2^f/f;D1::Cre^ mice (from data in A and B). (**D**), Sensitization ratios as in Fig. 3F (day 14, data from B, day 1 from A). (**E-H**) Same experiments as in A-D but in Pyk2^f/f^ (n = 14 mice) and Pyk2^f/f;A2A::Cre^ mice (n = 12). **E**, **F**, Locomotor activity of Pyk2^f/f^ and Pyk2^f/f;A2A::Cre^ mice after the first (**E**) and the second (**F**) injection of cocaine. (**G**), Pairwise comparison of the distance traveled 0–15 minutes following the first and the second injection in Pyk2^f/f^ and Pyk2^f/f;A2ACre^ mice (from data in **E** and **F**). (**H**), Sensitization ratios as in Fig. 3F in Pyk2^f/f^ and Pyk2^f/f;A2A::Cre^ mice. (**A-H**), Data are means ± SEM, indicated by a shaded area in A, B, E, and F, and horizontal bars in C-D and G-H. In A-C and E-G data were analyzed with 2-way ANOVA (see values in Results) followed by post-hoc Sidak’s multiple comparisons test, and in D and H by Mann and Whitney test. ns, not significant, *p < 0.05, **p < 0.01, ****p < 0.0001. See Supplementary Table 1 for detailed statistical analyses.

To determine which SPN population was involved in the consequences of Pyk2 deletion, we studied cocaine responses in Pyk2^f/f;D1::Cre^ and Pyk2^f/f;A2A::Cre^ mice (see Fig. 1D,E). Acute locomotor effects of the first injection of cocaine were decreased in Pyk2^f/f;D1::Cre^ as compared to Pyk2^f/f^ control mice (Fig. 5A, two-way ANOVA, genotype effect, F_1,729_ = 35.3, p < 10^−4^, see Supplementary Table 1 for details of all statistical analyses). The response of Pyk2^f/f;D1::Cre^ mice to the second injection of cocaine was also decreased as compared to matched controls, but was larger than after the first injection in both groups (Fig. 5B, two-way ANOVA, genotype effect, F_1,729_ = 47.5, p < 10^−4^). Paired analysis showed that both Pyk2^f/f^ and Pyk2^f/f;D1::Cre^ were sensitized (Fig. 5C, two-way ANOVA, injection effect: F_1,46_ = 42.6, p < 10^−4^; Sidak’s multiple comparisons post hoc tests: Pyk2^f/f^, t_46_ = 5.89, p < 10^−4^, Pyk2^f/f;D1::Cre^, t_46_ = 3.39, p = 0.0029).A similar sensitization ratio was observed in both genotypes (Fig. 5D, Mann-Whitney test, U_24,22_ = 228, p = 0.44). In contrast with the effect of Pyk2 deletion in D1 neurons, Pyk2^f/f;A2A::Cre^ mice and Pyk2^f/f^ mice displayed similar responses to the first (Fig. 5E, two-way ANOVA, genotype effect, F_1,432_ = 0.44, p = 0.51) and second injection (Fig. 5F, F_1,432_ = 2.85, p = 0.09). Both groups were sensitized (Fig. 5G, two-way ANOVA, injection effect: F_1,24_ = 38.68, p < 10^−4^; Sidak’s multiple comparisons post hoc tests: Pyk2^f/f^, t_24_ = 5.06, p < 10^−4^, Pyk2^f/f;A2a::Cre^, t_24_ = 3.79, p = 0.0018) and the sensitization ratios were not different (Fig. 5H, Mann-Whitney test, U_14,12_ = 79, p = 0.81). These results indicate that the absence of Pyk2 from D1R-expressing cells is responsible for a decreased locomotor response to cocaine, whereas deletion in A_2A_R-expressing cells has no effect in this test.

### Pyk2 knockout selectively blunts the acute locomotor effects of a D1R agonist but not a cholinergic antagonist

**Figure 6:**
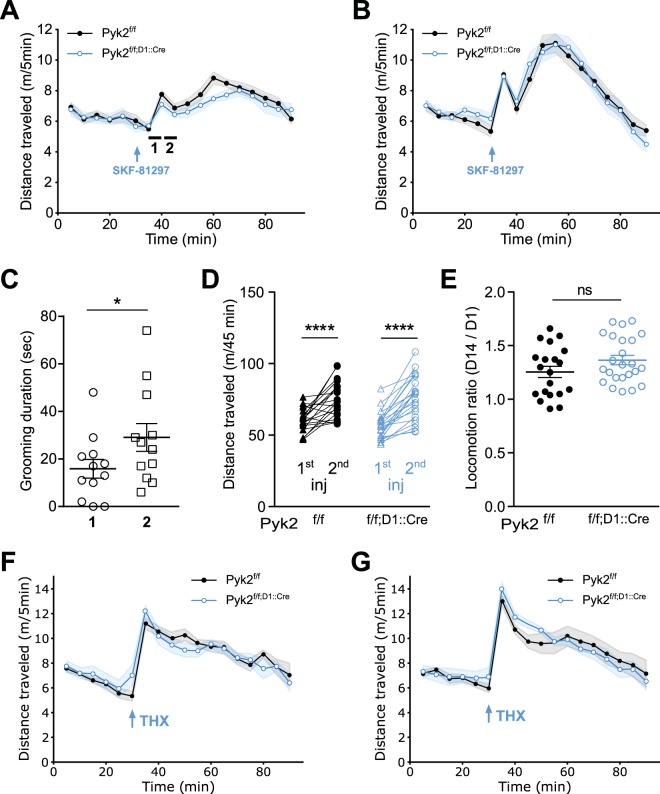
Selective deletion of Pyk2 in D1R-expressing cells blunts the acute locomotor response to a D1R agonist, but not to an anticholinergic agent. A-B, Locomotor activity of Pyk2^f/f^ (n = 20 mice) and Pyk2^f/f;D1::Cre^ mice (n = 27 mice) after a first (**A**) and a second (**B**, 13 days later) injection of the D1 agonist SKF-81297 (SKF, 3 mg/kg, arrow) injected 30 min after mice were placed in the open field. In A the locomotor activity was lower in Pyk2^f/f;D1::Cre^ mice than in Pyk2^f/f^ mice (2-way ANOVA, genotype effect p < 0.05 (Supplementary Table 1). (**C**), Time spent grooming was quantified as an index of stereotypies 35–40 min and 40–45 min (periods 1 and 2, respectively, indicated by an horizontal line in A) after the beginning of the recording in 6 randomly selected mice of each genotype. Since there was no difference between the two genotypes, data were pooled. (**D**), Pairwise comparison of the distances traveled during the 45 minutes following the first and the second SKF injections in Pyk2^f/f^ and Pyk2^f/f;D1::Cre^ mice (data from A and B). (**E**), Sensitization ratios, as in Fig. 3F, during the 45 min after SKF injection in the two genotypes (data from D). (**F-G)**, Specific deletion of Pyk2 in D1 neurons does not alter the locomotor responses to an anticholinergic drug, trihexyphenidine (THX). Locomotor activity of Pyk2^f/f^ (n = 12 mice) and Pyk2^f/f;D1::Cre^ mice (n = 13) after a first (**F**) and, 13 days later, a second (**G**) injection of THX (15 mg/kg i.p., arrow) injected 30 min after mice were placed in the open field. Data in A, B, D, F, and G were analyzed with 2-way ANOVA and post-hoc Sidak’s multiple comparisons test, those in C and E with two-tailed unpaired Students t-test. ns, not significant, *p < 0.05, ****p  <  10^−4^. See Supplementary Table 1 for detailed statistical analyses.

Since the consequences of the absence of Pyk2 on acute locomotor effects of cocaine appeared to result from Pyk2 deficit in D1 neurons, we examined whether the effects of D1R stimulation were altered. We used SKF-81297, a D1R selective agonist, known to increase locomotor activity^38–40^. The acute locomotor response to SKF-81297 was slightly reduced in Pyk2^f/f;D1::Cre^ mice as compared to Pyk2^f/f^ mice (Fig. 6A, genotype effect, F_1,801_ = 5.80, p = 0.016). These animals received a second injection of SKF-81297 13 days later that triggered a stronger locomotor response than the first injection, without difference between genotypes (Fig. 6B, genotype effect, F_1,752_ = 0.36, p = 0.55). Interestingly, both the first and second injection of SKF-81297 induced a biphasic effect on locomotor activity, not previously reported to our knowledge. Inspection of the videos suggested the existence of stereotypies at the trough between the two peaks of locomotor activity. Quantification of grooming duration showed no significant difference between the two genotypes but an increased time spent grooming 40–45 min after the beginning of the recording (i.e. 10–15 min post-injection) as compared to 35–40 min after the beginning of the recording (i.e. 15–20 min post-injection, Fig. 6C) strongly indicating that the presence of stereotypy accounted for the transiently decreased locomotion. As reported in previous studies^41–43^, a sensitization of the response was observed following the second injection of SKF-81297. We analyzed the sensitization during the 45 minutes following injection (Fig. 6D and E). The distance traveled after the second injection of SKF-81297 was increased (Fig. 6D, two-way ANOVA, injection effect: F_1,42_ = 83.86, p < 10^−4^; Sidak’s multiple comparisons post hoc tests: Pyk2^f/f^, t_42_ = 5.19, p < 10^−4^, Pyk2^f/f;D1::Cre^, t_42_ = 7.90, p < 10^−4^) but the sensitization ratios did not differ between genotypes (Fig. 6E, Student’s t test, t_42_ = 1.61, p = 0.11).

We then sought to determine whether Pyk2 could decrease any kind of drug-induced locomotor activity by testing the effects of trihexyphenidine (THX), an anticholinergic substance that increases locomotor activity^44^. There was no difference in locomotor activity between Pyk2^f/f;D1::Cre^ and Pyk2^f/f^ mice following the first (Fig. 6F, two-way ANOVA, genotype effect: F_1,414_ = 0.004, p = 0.95) or second injection of THX (Fig. 6G, F_1,396_ = 0.44, p = 0.51). No sensitization of the response was observed in either genotype. These results indicate that deletion of Pyk2 in D1R-expressing neurons has a slight effect on the locomotor effects of the first injection of a D1 agonist, but not of an anticholinergic agent. In addition, we provide evidence of a biphasic effect of SKF-81297 and a sensitization of both components upon a second administration of the drug.

### Pyk2 knockout does not alter cocaine-induced conditioned place preference

**Figure 7:**
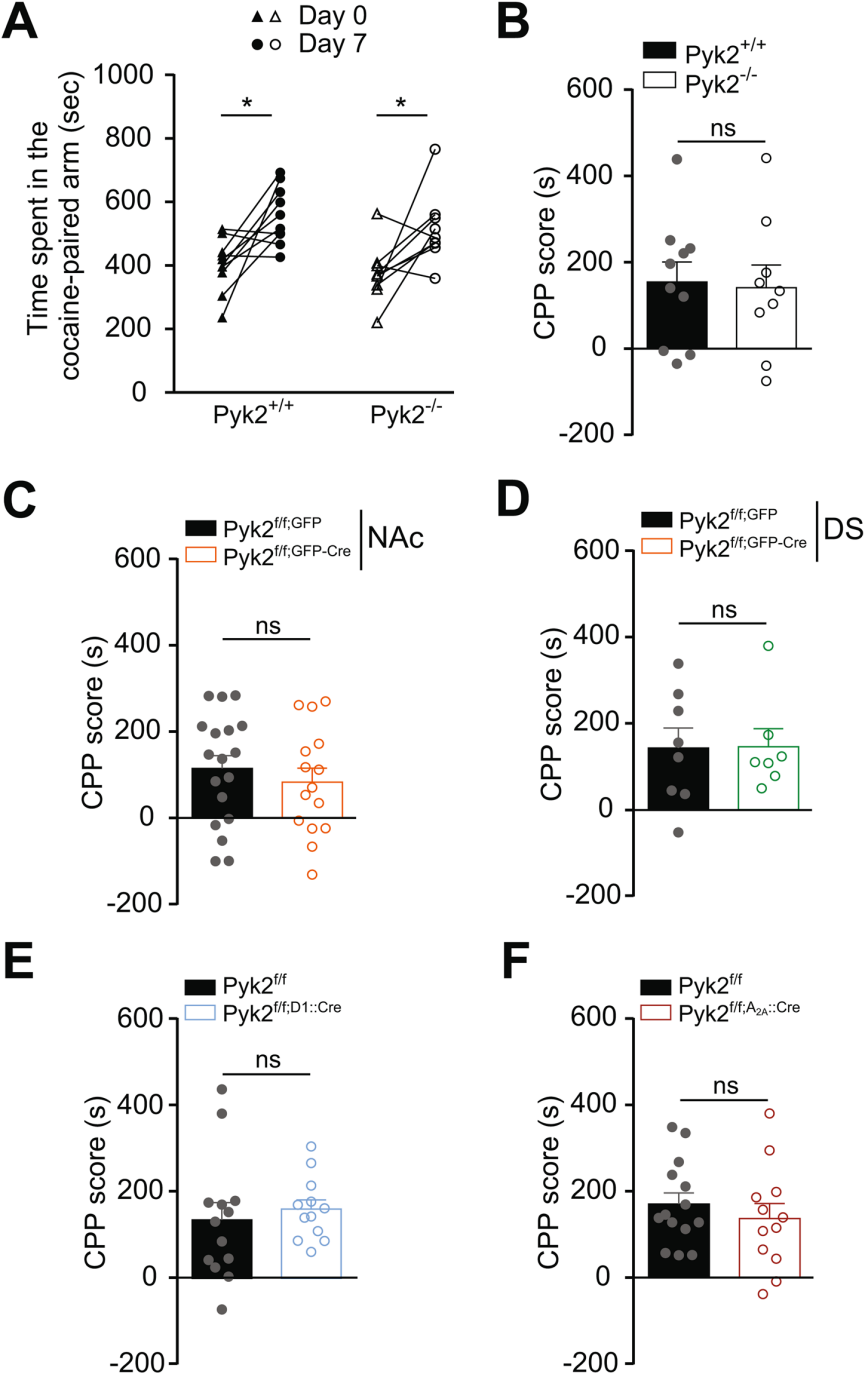
Pyk2 deletion does not alter cocaine-conditioned place preference. For each group of Pyk2 mutant mice and their respective matched controls, the time spent in the cocaine-paired arm before (Day 0) and after (Day 7) cocaine (15 mg/kg) conditioning was measured (see Materials and Methods). (**A**), Time spent in the cocaine-paired arm at days 0 and 7 by Pyk2^+/+^ (n = 10 mice) and Pyk2^-/-^ mice (n = 9). (**B**), The CPP score was calculated for each Pyk2^+/+^ and Pyk2^-/-^ mouse in A as the excess time spent in the cocaine-paired arm. (**C**), CPP score as in B for in mice injected in the NAc with AAV expressing GFP or Cre and GFP (Pyk2^f/f;NAc,GFP^, n = 18, and Pyk2^f/f;NAc,GFP-Cre^ mice, n = 15). (**D**), Same as in C, except that the injection was in the DS (Pyk2^f/f;DS,GFP^, n = 8, and Pyk2^f/f;DS,GPF-Cre^, n = 7). (**E**), Same as in B, in Pyk2^f/f^ (n = 13) and Pyk2^f/f;D1::Cre^ (n = 12) mice. (**F**), Same as in B, in Pyk2^f/f^ (n = 14) and Pyk2^f/f;A2A::Cre^ (n = 12) mice. B-F Bars are means + SEM. Analysis was done with 2-way ANOVA in A followed by Sidak’s test, and with unpaired Mann-Whitney test in B-F (see Results). See Supplementary Table 1 for detailed statistical analyses.

We then evaluated in Pyk2 mutant mice the rewarding properties of cocaine, which involve D1R^45–47^. We measured cocaine-induced CPP in wild-type and mutant mice (Fig. 7). The time spent in the cocaine-paired arm after conditioning was similarly increased in Pyk2^-/-^ and Pyk2^+/+^ mice (Fig. 7A, two-way ANOVA, injection effect: F_1,17_ = 17.90, p = 0.0006, Sidak’s multiple comparisons post hoc tests, Pyk2^+/+^, t_17_ = 3.21, p = 0.01, Pyk2^-/-^, t_17_ = 2.79, p = 0.025). The CPP score did not differ between Pyk2^-/-^ and Pyk2^+/+^ mice (Fig. 7B, Mann-Whitney test, U_10,9_ = 39, p = 0.96), demonstrating that mice underwent efficient CPP in the absence of Pyk2. There was also no effect on CPP scores of the specific deletion of Pyk2 in the NAc (Fig. 7C, U_18,15_ = 112, p = 0.41), in the DS (Fig. 7D, U_8,7_ = 27, p = 0.95) or in D1R- (Fig. 7E, U_13,12_ = 60, p = 0.35) and A_2A_R-expressing SPNs (Fig. 7F, U_14,12_ = 69, p = 0.45). The lack of modification of CPP scores in any of the mutant groups as compared to their respective controls, showed that Pyk2 was not necessary for CPP in our conditions.

### The absence of Pyk2 in D1 neurons decreases cocaine-induced GluN2B phosphorylation but does not alter ERK activation

**Figure 8:**
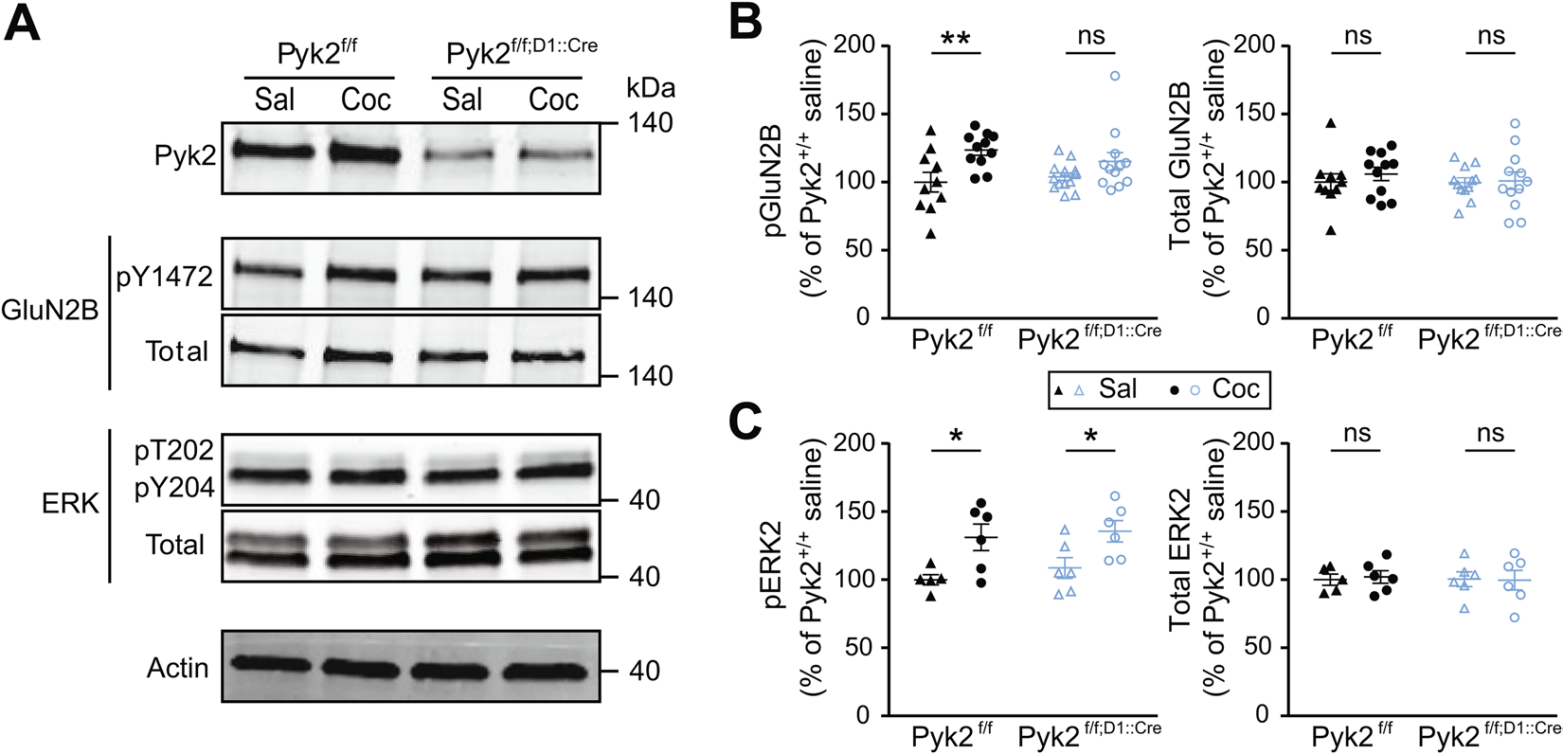
Specific deletion of Pyk2 in D1 neurons decreases cocaine effects on GluN2B phosphorylation but not ERK. (**A**), Immunoblotting analysis of Pyk2, GluN2B phosphorylated at Tyr-1472 (pY1472), ERK phosphorylated at Thr-202 and Tyr-204 (pT202/pY204), total levels (Total) of GluN2 and ERK, and actin as loading control in 3-month Pyk2^f/f^ and Pyk2^f/f;D1::Cre^ mice, 10 min after saline (Sal) or cocaine (20 mg/kg, Coc) i.p. injection. (**B**), Densitometry quantification of phospho (left) and total (right) GluN2B levels normalized to actin. (**C**), Densitometry quantification of phosphorylated (left) and total (right) ERK levels normalized to actin. (**B**,**C**), Means are indicated ± SEM. Analyses with two-way ANOVA, followed by Sidak’s multiple comparisons post hoc tests. ns, not significant, *p < 0.05, **p < 0.01. B, n = 10–13 mice/group. C, n = 5–6 mice/group. See Supplementary Table 1 for detailed statistical analyses and Supplementary Figure 4 for full length blots.

Cocaine injection was reported to increase GluN2B phosphorylation at Tyr-1472 and to activate SFKs^8^. To evaluate the role of Pyk2 in this response we compared the effects of cocaine on Tyr1472-GluN2B phosphorylation in Pyk2^f/f;D1::Cre^ and in Pyk2^f/f^ mice. In Pyk2-mutant mice, 10 min after cocaine injection, the increase in pTyr1472-GluN2B was blunted and not significant (Fig. 8A upper panels and B, pGluN2B, two-way ANOVA, drug effect, F_1,42_ = 10.89, p = 0.002, Sidak’s multiple comparisons, Pyk2^f/f^, t_42_ = 3.03, *p* = 0.008, Pyk2^f/f;D1::Cre^, t_42_ = 1.58, p = 0.23; total GluN2B, drug effect: F_1,41_ = 0.48, p = 0.49). This minor alteration of GluN2B phosphorylation reflects a role of Pyk2 in the phosphorylation of NMDA receptor at Tyr-1472, either directly or through recruitment of SFKs. This response was previously reported to contribute to the activation of the ERK pathway^8^. However, the absence of Pyk2 in D1 SPNs was not sufficient to prevent the phosphorylation of ERK (Fig. 8A lower panels and C, pERK2, two-way ANOVA, drug effect: pGluN2B, F_1,19_ = 13.7, p = 0.0015, Sidak’s multiple comparisons, Pyk2^f/f^, t_19_ = 2.74, *p* = 0.026, Pyk2^f/f;D1::Cre^, t_19_ = 2.49, p = 0.044; total ERK, drug effect: F_1,19_ = 0.011, p = 0.92). The lack of alteration in ERK phosphorylation indicated that compensatory signaling mechanisms^7^ were efficient to activate ERK in the mutant mice.

## Discussion

In this study, we show the involvement of the non-receptor tyrosine kinase Pyk2 in the acute locomotor response to cocaine. We demonstrate that complete knockout of Pyk2 or its specific deletion in the NAc or in D1R-expressing neurons decreases acute cocaine-induced hyperlocomotion. Acute locomotor response to a selective D1 agonist but not to a cholinergic antagonist was also slightly altered. These results indicate a role of Pyk2 located in D1R-expressing neurons of the NAc in the D1R-induced acute locomotor response. In contrast, no alteration was observed in locomotor sensitization or cocaine-induced CPP.

Our immunofluorescence data in the striatum agree with previous results suggesting the enrichment of Pyk2 in the ventral part of the striatum^27^. We also provide evidence for a relative enrichment in D1R-expressing SPNs, which form the ‘direct pathway’^48^, and in the matrix compartment of the striatum, which plays a specific yet incompletely characterized role in striatal function^49^. The dorsal striatum is implicated in the control of movement and in learning skilled motor tasks^50,51^. We assessed motor coordination of mice with accelerating rotarod training^52^, which can be impaired by alteration of several brain areas, including basal ganglia, motor cortex, and cerebellum^53^. We did not observe any difference between any of the complete or conditional Pyk2 mutant mice we generated and their respective controls, suggesting that Pyk2 is not required for the motor function of the dorsal striatum or other brain regions. Pyk2 appears to be more expressed in the NAc, which is a key component in brain circuitry underlying drug-evoked behaviors^54,55^. We demonstrated that tyrosine phosphorylation of Pyk2 in the striatum was increased following a single injection of cocaine. Pyk2 is activated by Ca^2+^-induced autophosphorylation of Tyr-402, followed by recruitment of SFKs which in turn phosphorylate Pyk2 on other tyrosine residues, although in some cells activation of SFKs can be the triggering mechanism (see^17^ for a review). Cocaine injection in naïve mice increases the concentration of calcium in D1 SPNs^56^ providing a possible basis for Pyk2 activation. In addition, cocaine-induced activation of Fyn has been reported in the striatum and proposed to be involved in NMDA receptors regulation and their synergism with D1R to activate the ERK pathway^8^. Although these results suggested a possible role of Pyk2 in ERK activation, ERK phosphorylation was still observed in Pyk2-deficient mice, underlining the redundancy in the mechanisms controlling the ERK pathway.

Concerning the functional consequences of Pyk2 knock-out, we found that the acute cocaine locomotor response was decreased, whereas locomotor sensitization and CPP, which are known to involve synaptic plasticity and ERK activation (see^7^ for a review), were not altered. Although our results did not indicate effects of Pyk2 mutation on other acute effects of cocaine or D1 receptor agonist such as stereotyped behavior, a more detailed study would be necessary to evaluate minute specific alterations. The acute locomotor response was also impaired in mice specifically lacking Pyk2 in the NAc or in D1R-expressing neurons, but not in those devoid of Pyk2 in the DS or in A_2A_R-expressing neurons. In mice lacking Pyk2 in D1 neurons a decreased locomotion was even observed after the second cocaine injection. This combination of results implicates an alteration induced by the absence of Pyk2 in the D1R-expressing neurons of the NAc. This conclusion is in line with the role of these neurons in the acute locomotor effects of psychostimulants^57–59^. A decreased locomotor response without alteration in locomotor sensitization or CPP was previously observed in G*α*olf heterozygous (*Gnal*^+/−^) mutant mice in which cAMP production is decreased^60^. These similarities could indicate a modulation of the D1R/G*α*olf/cAMP pathway by Pyk2, a hypothesis supported by the attenuated locomotor response to the D1 agonist SKF-81297 in Pyk2^f/f;D1::Cre^ mice. However, G*α*olf levels were not changed in the mutant mice (see Fig. 1H and I) and phosphorylation of cAMP-dependent protein kinase substrates was not altered (de Pins and Girault, unpublished observation). The only effect we observed was a blunting of GluN2B receptor phosphorylation at Tyr1472, in agreement with the role of Pyk2 in the phosphorylation of NMDAR^11^, but unlikely to explain by itself the effects on locomotor activity. Therefore we hypothesize that Pyk2 is also implicated in the regulation of other signaling mechanisms important for D1R-mediated regulation of locomotor activity. Further work will be necessary to explore this hypothesis. Our study clearly identifies the significant but circumscribed contribution of Pyk2 in NAc D1 neurons to the acute locomotor effects of cocaine. It raises the question of its role in other dopamine-mediated actions and opens novel avenues for investigating the role of tyrosine phosphorylation and non-receptor tyrosine kinases signaling in striatal neurons.

## Materials and Methods

### Animals

Floxed Pyk2 mice (Pyk2^f/f^) were generated by the insertion of LoxP sequences (Gen-O-way, Lyon, France) surrounding *PTK2B* exons 15b-18 coding for the kinase domain^61^. Homologous recombination was carried out in C57/Bl6 embryonic stem cells and germline transmission of the mutated allele was achieved in the same background. Floxed Pyk2 mice were initially bred to Cre-deleter mice to generate constitutive knockout mice, or to *Drd1::Cre* mice, Tg(Drd1a-cre)EY262Gsat^62^, or *Adora2a::Cre* mice, Tg(Adora2a-cre)2MDkde^63^, to generate conditional KO mice (Pyk2^f/f;D1::Cre^ and Pyk2^f/f;A2A::Cre^, respectively). Mice were housed at 19–22 °C with 40–60% humidity, under a 12:12 h light/dark cycle, and had ad libitum access to food and water. Animal experiments and handling were in accordance with ethical guidelines of Declaration of Helsinki and NIH, (1985-revised publication no. 85–23, European Community Guidelines), and French Agriculture and Forestry Ministry guidelines for handling animals (decree 87849, licence A 75-05-22) and approval of the Charles Darwin ethical committee APAFIS#8861-2016111620082809. Mice used in this study were 3-6-month-old males.

### Viral vectors and stereotaxic injection

For deletion of Pyk2 in the NAc or dorsal striatum (DS), 3-month Pyk2^f/f^ mice were stereotaxically injected with AAV expressing an enhanced green fluorescent protein (EGFP) Cre recombinase fusion protein (AV-9-PV2521, AAV9.CamKII.HI.eGFP-Cre.WPRE.SV40, Perelman School of Medicine, University of Pennsylvania, USA), here referred to as AAV-GFP-Cre. As control, we injected AAVs expressing GFP (AV-9-PV1917, AAV9.CamKII0.4.eGFP.WPRE.rBG, same source), referred to as AAV-GFP. Following anesthesia with pentobarbital (30 mg kg^−1^), we performed bilateral stereotaxic injections of AAV-GFP or AAV-GFP-Cre (2.6 × 10^9^ GS per injection) in the NAc at the following coordinates^64^ from the bregma (millimeters), anteroposterior, 1.3, lateral, ±1.3, and dorsoventral, −4.5 or in the DS, anteroposterior, 0.9, lateral, ±1.5, and dorsoventral, −2.75. AAV injection was carried out in 2 min. The cannula was left in place for 5 min for complete virus diffusion before being slowly pulled out of the tissue. Mice were placed on a warm plate for 2 h after surgery, received a subcutaneous injection of a non-steroidal anti-inflammatory drug (meloxicam, 2 mg/kg) during 3 days, and allowed to recover for 3 weeks before starting behavioral experiments.

### Behavioral experiments

#### Rotarod

4-month mice were trained at accelerating speed (4–40 rpm in 5 min), with four sessions per day for three consecutive days and the latency to fall was recorded.

#### Locomotor activity

Mice were placed either in a open-field chamber (50 cm × 50 cm, L x W) for cocaine response or in a 20-cm diameter cylinder for SKF-81297 [SKF] or trihexyphenidyl [THX] response. After 30 minutes, mice were i.p. injected with cocaine (20 mg/kg), SKF (3 mg/kg), or THX (15 mg/kg) and placed back in the chamber for 1 hour. Locomotion was recorded using an overhead digital camera. The distance traveled was measured in 5-min bins using EthoVision software (Noldus, Wageningen, the Netherlands).

#### Rearing and grooming

These two behaviors were manually scored during visualization of the videos recorded to measure locomotor activity during the period indicated in the Results.

#### Conditioned place preference

Conditioned place preference (CPP) was performed in two compartments of a Y-shaped maze (Imetronic, Pessac, France) with different wall textures and visual cues as follows. (i) Pretest: day-0, mice were placed in the center of the apparatus and allowed to explore freely both compartments for 20 min. The time spent in each compartment was recorded and the preferred and un-preferred compartments deduced for every mouse. (ii) Conditioning: day-1, mice were injected with saline and placed immediately in the preferred compartment for 15 min. The next day, they were placed in the other closed compartment after cocaine injection (15 mg/kg). This was repeated twice (3 saline-, 3 cocaine-pairings in total). (iii) Test: time spent in each compartment was measured on day-7 during 20 min. The CPP-score was calculated as the time spent in the cocaine-paired compartment during the test minus the time spent in this compartment during the pre-test.

### Tissue preparation and immunofluorescence

Mice were euthanized, brains rapidly dissected and divided at the midline; one hemisphere was drop-fixed in 40 g/L paraformaldehyde (PFA) for 24 hours. The other hemisphere was flash-frozen using CO_2_ pellets and stored at −80 °C. Following PFA fixation, 30-µm-thick sections were cut with a Vibratome (Leica, Wetzlar, Germany). Sections were incubated overnight with primary antibodies at 4 °C: chicken anti-GFP (1:500; #A10262, Thermo Fisher, Waltham, MA, USA), mouse anti-calbindin (1:1000; #300, Swant, Switzerland), rabbit anti-Pyk2 (1:500; #P3902, Sigma-Aldrich). After rinses in TBS (150 mM NaCl, 20 mM Tris-HCl, pH 7.5), sections were incubated for 45 min with secondary antibodies (1:400 dilution; Cy3 goat anti-rabbit and/or Alexa488 goat anti-mouse antibodies; Jackson ImmunoResearch, West Grove, PA, USA). Nuclei were labelled with DAPI-containing Vectashield (Vector Laboratories, Burlingame, CA, USA). Pyk2 and GFP overall distribution was imaged with a DM6000–2 microscope (Leica). Calbindin and Pyk2 images were acquired with a Leica Confocal SP5-II (63× numerical aperture lens, 5× digital zoom, 1-Airy unit pinhole, 4-frame averaging per *z*-step, *z*-stacks every 2 μm, 1024 × 1024 pixel resolution). Images were analyzed with Icy open source software (https://icy.bioimageanalysis.org)^65^.

### Immunobloting

For the analysis of striatal proteins untreated mice were euthanized by cervical dislocation, striata dissected out, frozen using CO_2_ pellets and stored at −80 °C until use. For pharmacological responses mice were i.p. injected with cocaine (20 mg/kg) or saline and placed in a 43 cm × 27 cm cage. After 10 minutes, mice were euthanized and heads were dipped in liquid nitrogen for 12 seconds. The frozen heads were cut into 210-μm-thick slices with a cryostat, and 10 frozen microdisks (1.4 mm diameter) were punched out bilaterally from the striatum and stored at −80 °C until use. Tissue samples were sonicated in 10 g/L SDS and 1 mM sodium orthovanadate in water, and placed at 100 °C for 5 min. Extracts (15 μg protein) were separated by SDS–PAGE and transferred to nitrocellulose membranes (GE Healthcare, Chicago, IL, USA). Membranes were blocked in TBS-T (0.5 ml l^−1^ Tween 20) with 30 g/L BSA. Immunoblots were probed with the following antibodies (all diluted 1:1000): rabbit polyclonal antibodies: Pyk2 (#P3902, Sigma-Aldrich), PSD-95 (#3450, Cell Signaling Technology, Danvers, MA, USA), phosphoY1472-GluN2B (#10009761, Cayman chemical, Ann Arbor, MI, USA), GluN2B (#06–600, Merck KGaA, Darmstadt, Germany), GluN2A (#05–901 R, Merck KGaA), phosphoT202/Y204-ERK1/2 (#9101, Cell Signaling Technologies), ERK1/2 (#9102, Cell Signaling Technologies), Gαolf (produced as described^66^), synapsin 1 (1:500; G-486, gift from Pr. Greengard, The Rockefeller University), tyrosine hydroxylase (#AB1541, Merck KGaA), and STEP (#9069, Cell Signaling Technology) mouse monoclonal antibodies: Pyk2 (#3480, Cell Signaling Technology), DARPP-32 (1:5000; mAb6, gift from Pr. Greengard) and phosphotyrosine (#05–947, Cell Signaling Technology) chicken polyclonal antibody: tyrosine hydroxylase (#TH, Aves Labs, Tigard, OR, USA). All blots were incubated with the primary antibody overnight at 4 °C by shaking in TBS. After several washes in TBS-T, blots were incubated with secondary anti-rabbit, anti-mouse or anti-chicken IgG DyLight™ 800 or 680 conjugated antibodies (1:10000; Rockland Immunochemicals, Pottstown, PA, USA). Secondary antibody binding was detected by Odyssey infrared imaging apparatus (Li-Cor Inc., Lincoln, NE, USA). For loading control a mouse monoclonal antibody for β-actin was used (1:5000; #A5441, Sigma-Aldrich).

### Immunoprecipitation

Mice were i.p. injected with cocaine (20 mg/kg) and placed in a 43 cm × 27 cm cage. After 10 minutes, mice were euthanized and heads dipped in liquid nitrogen for 5 seconds. The striatum was dissected out, lysed by sonication in 250 μl NP-40 lysis buffer [150 mM NaCl, 50 mM Tris-HCl, pH 8.0, 10 mM NaF, 1% NP40 (v/v) supplemented with 1 mM sodium orthovanadate, phosphatase inhibitor (PhosSTOP, Roche, Basel, Switzerland) and protease inhibitor (Complete, Roche)]. Lysates were centrifuged for 20 min at 20,937 g (4 °C). Protein A-Sepharose beads (GE Healthcare) were pre-cleared by mixing with 14% Sephacryl S-100 (v/v) (GE Healthcare) and saturated with BSA (25 g/L). The beads were then mixed for 1 hour at 4 °C with rabbit polyclonal anti-Pyk2 antibody (1.7% v/v) (#P3902, Sigma-Aldrich, St. Louis, MO, USA) prior incubation overnight at 4 °C with supernatant. The beads were washed three times, resuspended in Laemmli loading buffer, heated at 100 °C for 10 min, and subjected to SDS-PAGE.

### Statistical analysis

Analyses were done using Prism version 6.00 for Windows (GraphPad Software, La Jolla, CA, USA). Data are expressed as means +SEM. Normal distribution was tested with d’Agostino and Pearson omnibus, Shapiro-Wilk, and Kolmogorov-Smirnov tests. If no difference from normality was detected, statistical analysis was performed using two-tailed Student’s *t* test or ANOVA and Sidak’s post-hoc test. Otherwise or when n was <7, non-parametric two-tailed Mann-Whitney test was used. p < 0.05 was considered as significant. Number of experimental points and animals, and results of statistical analyses are presented in Supplementary Table 1.

## Supplementary information

Supplementary Information.
