# Supplementary material for: The non-receptor tyrosine kinase Pyk2 modulates acute locomotor effects of cocaine in D1 receptor-expressing neurons of the nucleus accumbens
Source: Sci Rep. 2020 Apr 20;10:6619. doi: 10.1038/s41598-020-63426-5 (PMC7170924; doi:10.1038/s41598-020-63426-5)
Supplement: Supplementary file 1 — Supplementary Information. [file 41598_2020_63426_MOESM1_ESM.pdf]

Supplementary Material

The non-receptor tyrosine kinase Pyk2 modulates acute locomotor effects of cocaine in D1 receptor-expressing neurons of the nucleus accumbens

Benoit de Pins[1,2,3], Enrica Montalban[1,2,3], Peter Vanhoutte[2,4,5], Albert Giralt[1,2,3,*], and Jean-Antoine Girault[a,1,2,3,*]

[1]Inserm UMR-S 1270, Paris 75005, France.

[2]Sorbonne Université, Faculty of Sciences and Engineering, Paris 75005, France.

[3]Institut du Fer à Moulin, Paris 75005, France.

[4]Inserm UMR-S 1130, Neurosciences Paris Seine, Paris 75005, France.

[5]CNRS UMR 8246, Paris 75005, France.

*equal contribution

[a] Corresponding author, e-mail: jean-antoine.girault@inserm.fr

Current addresses:

- Benoit de Pins, Department of Plant and Environmental Sciences, Weizmann Institute of Science, Rehovot 7610001, Israel.

- Enrica Montalban, BFA - *Unité de Biologie Fonctionnelle et Adaptative* - CNRS UMR 8251, Paris University, Paris 75205, France

- Albert Giralt, Departament de Biomedicina, Facultat de Medicina, Institut de Neurociències, Universitat de Barcelona, Institut d'Investigacions Biomèdiques August Pi i Sunyer (IDIBAPS), Barcelona 08036, Spain and Centro de Investigación Biomédica en Red sobre Enfermedades Neurodegenerativas (CIBERNED), Madrid 28031, Spain.

Content

- **Supplementary Table 1:** Detailed statistical analyses results
- **Supplementary Figure 1:** Effects of cocaine on stereotyped behavior in Pyk2$^{+/+}$ and Pyk2$^{-/-}$ mice
- **Supplementary Figure 2:** Full length blots from Fig. 1DEF
- **Supplementary Figure 3:** Full length blots from Fig. 1H and 3A
- **Supplementary Figure 4:** Full length blots from Fig. 8A

Figure 1

Figure	Variable	Group	n mice	n data points	Failed normality test	Mean	SEM	Statistical analysis	Comparison	DF	Test value	*P* value
1A	Pyk2 IF (a.u.)	Dorsal Striatum (DS)	3	18	No	1 143	20	Student's t-test two-tailed unpaired	DS vs Nac	34	t = 4.3	0.0001
		Nucleus accumbens (Nac)	3	18	No	1 315	35					
1C	Pyk2 IF (a.u.)	Matrix	3	20	No	2 910	41	Mann Whitney's test two-tailed	Matrix vs Patch		U = 12	< 0.0001
		Patch	3	20	Yes	2 396	59					
1D	Pyk2 level (% of +/+)	Pyk2 f/f	11	11	Yes	100	4	Mann Whitney's test two-tailed	Pyk2 f/f vs Pyk2 f/f; D1::Cre		U = 0	< 0.0001
		Pyk2 f/f; D1::Cre	11	11	No	33	3					
1E	Pyk2 level (% of +/+)	Pyk2 f/f	7	7	No	100	8	Mann Whitney's test two-tailed	Pyk2 f/f vs Pyk2 f/f; A2a::Cre		U = 4	0.03
		Pyk2 f/f; A2A::Cre	5	5	No	64	3					
1G	Pyk2 (% of +/+)	Pyk2+/+	6	6	No	100	6	Mann Whitney's test two-tailed	Pyk2+/+ vs Pyk2-/-		U = 0	0.0022
		Pyk2-/-	6	6	No	1	0					
	GluN2A (% of +/+)	Pyk2+/+	6	6	No	100	6	Mann Whitney's test two-tailed	Pyk2+/+ vs Pyk2-/-		U = 13	0.48
		Pyk2-/-	6	6	No	108	7					
	GluN2B total (% of +/+)	Pyk2+/+	9	9	No	100	4	Mann Whitney's test two-tailed	Pyk2+/+ vs Pyk2-/-		U = 68	0.19
		Pyk2-/-	22	22	Yes	118	7					
	pY1472GluN2B (% of +/+)	Pyk2+/+	9	9	No	100	4	Mann Whitney's test two-tailed	Pyk2+/+ vs Pyk2-/-		U = 93	0.81
		Pyk2-/-	22	22	No	104	5					
	PSD-95 (% of +/+)	Pyk2+/+	6	6	No	100	4	Mann Whitney's test two-tailed	Pyk2+/+ vs Pyk2-/-		U = 24	> 0.99
		Pyk2-/-	8	8	Yes	106	8					
1I	Gαolf	Pyk2+/+	6	6	No	100	5	Mann Whitney's test two-tailed	Pyk2+/+ vs Pyk2-/-		U = 17	0.90
		Pyk2-/-	6	6	No	101	3					
	DARPP-32	Pyk2+/+	6	6	No	100	5	Mann Whitney's test two-tailed	Pyk2+/+ vs Pyk2-/-		U = 11	0.31
		Pyk2-/-	6	6	No	106	4					
	Synapsin I	Pyk2+/+	6	6	No	100	3	Mann Whitney's test two-tailed	Pyk2+/+ vs Pyk2-/-		U = 15	0.44
		Pyk2-/-	8	8	No	95	4					
	TH	Pyk2+/+	6	6	No	100	6	Mann Whitney's test two-tailed	Pyk2+/+ vs Pyk2-/-		U = 12	0.39
		Pyk2-/-	8	8	No	92	5					

Figure 2

Figure	Variable	Group	n mice	n data points	Statistical analysis	Comparison	DF	Test value	*P* value
**2A**	Latency to fall (s)	Pyk2+/+	9	9	Two-way ANOVA	Interaction	11 ; 191	F = 0.39	0.96
		Pyk2-/-	9	9		Trial number	11 ; 191	F = 14.03	**< 0.0001**
						Genotype	1 ; 191	F = 2.57	0.11
**2B**	Latency to fall (s)	Pyk2f/f ; NAc GFP	17	17	Two-way ANOVA	Interaction	11 ; 345	F = 0.44	0.94
		Pyk2f/f ; NAc Cre, GFP	14	14		Trial number	11 ; 345	F = 12.20	**< 0.0001**
						Genotype	1 ; 345	F = 1.83	0.18
**2C**	Latency to fall (s)	Pyk2f/f ; DS GFP	8	8	Two-way ANOVA	Interaction	11 ; 155	F = 0.70	0.74
		Pyk2f/f ; DS Cre, GFP	7	7		Trial number	11 ; 155	F = 4.99	**< 0.0001**
						Genotype	1 ; 155	F = 1.07	0.30
**2D**	Latency to fall (s)	Pyk2f/f	14	14	Two-way ANOVA	Interaction	11 ; 298	F = 0.59	0.84
		Pyk2f/f ; D1::Cre	9	9		Trial number	11 ; 298	F = 22.46	**< 0.0001**
						Genotype	1 ; 298	F = 0.022	0.88
**2E**	Latency to fall (s)	Pyk2f/f	14	14	Two-way ANOVA	Interaction	11 ; 286	F = 0.27	0.99
		Pyk2f/f ; A2A::Cre	12	12		Trial number	11 ; 286	F = 14.47	**< 0.0001**
						Genotype	1 ; 286	F = 0.84	0.36

Figure 3

Figure	Variable	Group	n mice	n data points	Failed normality test	Mean	SEM	Statistical analysis	Comparison	DF	Test value	*P* value
3B	pTyr/PYK2	Saline	7	7	No	100	9	Student's t-test two-tailed unpaired	Saline vs Cocaine	12	t = 2.49	**0.029**
		Cocaine	7	7	No	161	23					
3C	Distance traveled (m/5 min)	Pyk2+/+	10	10				Two-way ANOVA	Interaction	17 ; 306	F = 2.44	**0.0014**
		Pyk2-/-	9	9					Time	17 ; 306	F = 20.43	**< 0.0001**
									Genotype	1 ; 306	F = 39.05	**< 0.0001**
3D	Distance traveled (m/5 min)	Pyk2+/+	10	10				Two-way ANOVA	Interaction	17 ; 306	F = 0.43	0.98
		Pyk2-/-	9	9					Time	17 ; 306	F = 40.18	**< 0.0001**
									Genotype	1 ; 306	F = 1.59	0.21
3E	Distance traveled (m/15 min)	1st injection - Pyk2+/+	10	10		13 147	1 164	Two-way ANOVA Repeated measures	Interaction	1 ; 17	F = 0.90	0.36
		2nd injection - Pyk2+/+	10	10		16 230	1 384		Genotype	1 ; 17	F = 0.78	0.39
		1st injection - Pyk2-/-	9	9		10 885	1 264		Injection	1 ; 17	F = 25.49	**< 0.0001**
		2nd injection - Pyk2-/-	9	9		15 390	1 568		Subjects (matching)	17 ; 17	F = 5.44	**0.0005**
								Sidak's multiple comparisons test	Pyk2+/+, 1st inj vs 2nd inj	17	t = 2.98	**0.017**
								Sidak's multiple comparisons test	Pyk2-/-, 1st inj vs 2nd inj	17	t = 4.13	**0.0014**
3F	Locomotor response ratio, Day 14 / Day 1	Pyk2+/+	10	10	No	128	10	Student's t-test two-tailed unpaired	Pyk2+/+ vs Pyk2-/-	17	t = 1.24	0.23
		Pyk2-/-	9	9	No	145	8					

Figure 4

Figure	Variable	Group	n mice	n data points	Failed normality test	Mean	SEM	Statistical analysis	Comparison	DF	Test value	*P* value
4B	Distance traveled (m/5 min)	Pyk2f/f ; NAc GFP	11	11				Two-way ANOVA	Interaction	17 ; 342	F = 2.85	0.0002
		Pyk2f/f ; NAc Cre, GFP	10	10					Time	17 ; 342	F = 10.15	< 0.0001
									Cre effect	1 ; 342	F = 12.94	0.0004
4C	Distance traveled (m/5 min)	Pyk2f/f ; NAc GFP	11	11				Two-way ANOVA	Interaction	17 ; 342	F = 1.94	0.094
		Pyk2f/f ; NAc Cre, GFP	10	10					Time	17 ; 342	F = 22.16	< 0.0001
									Cre effect	1 ; 342	F = 0.015	0.90
4D	Distance traveled (m/15 min)	1st inj. Pyk2f/f ; NAc GFP	11	11		106	12	Two-way ANOVA Repeated measures	Interaction	1 ; 19	F = 0.84	0.37
		2nd inj. Pyk2f/f ; NAc GFP	11	11		137	12		Cre effect	1 ; 19	F = 6.99	0.016
		1st inj. Pyk2f/f ; NAc Cre, GFP	11	11		61	11		Injection	1 ; 19	F = 20.05	0.0003
		2nd inj. Pyk2f/f ; NAc Cre, GFP	10	10		107	12		Subjects (matching)	19 ; 19	F = 2.71	0.018
								Sidak's multiple comparisons test	Pyk2f/f ; NAc GFP 1st inj vs 2nd inj	19	t = 2.58	0.036
								Sidak's multiple comparisons test	Pyk2f/f ; NAc Cre, GFP 1st inj vs 2nd inj	19	t = 3.73	0.003
4E	Locomotor response ratio, Day 14 / Day 1	Pyk2f/f ; NAc GFP	11	11	No	141	14	Mann Whitney's test two-tailed	Pyk2f/f ; NAc GFP vs NAc Cre, GFP		U = 39	0.28
		Pyk2f/f ; NAc Cre, GFP	10	10	Yes	234	57					
4G	Distance traveled (m/5 min)	Pyk2f/f ; DS GFP	8	8				Two-way ANOVA	Interaction	17 ; 234	F = 0.16	> 0.99
		Pyk2f/f ; DS Cre, GFP	7	7					Time	17 ; 234	F = 3.00	< 0.0001
									Cre effect	1 ; 234	F = 0.55	0.46
4H	Distance traveled (m/5 min)	Pyk2f/f ; DS GFP	8	8				Two-way ANOVA	Interaction	17 ; 234	F = 0.78	0.72
		Pyk2f/f ; DS Cre, GFP	7	7					Time	17 ; 234	F = 22.93	< 0.0001
									Cre effect	1 ; 234	F = 7.93	0.0053
4I	Distance traveled (m/15 min)	1st inj. Pyk2f/f ; DS GFP		8		79	10	Two-way ANOVA Repeated measures	Interaction	1 ; 13	F = 0.015	0.90
		2nd inj. Pyk2f/f ; DS GFP		8		137	14		Cre effect	1 ; 13	F = 0.11	0.75
		1st inj. Pyk2f/f ; DS Cre, GFP		7		85	17		Injection	1 ; 13	F = 26.55	0.0002
		2nd inj. Pyk2f/f ; DS Cre, GFP		7		140	10		Subjects (matching)	13 ; 13	F = 1.78	0.16
								Sidak's multiple comparisons test	Pyk2f/f ; DS GFP 1st inj vs 2nd inj	13	t = 3.86	0.004
								Sidak's multiple comparisons test	Pyk2f/f ; DS Cre, GFP 1st inj vs 2nd inj	13	t = 3.44	0.009
4J	Locomotor response ratio, Day 14 / Day 1	DS-GFP	8	8	No	188	26	Mann Whitney's test two-tailed	Pyk2f/f ; DS GFP vs DS Cre, GFP		U = 27	0.92
		DS-Pyk2-/-	7	7	Yes	225	66					

Figure 5

Figure	Variable	Group	n mice	n data points	Failed normality test	Mean	SEM	Statistical analysis	Comparison	DF	Test value	*P* value
5A	Distance traveled (m/5 min)	Pyk2f/f	24	24				Two-way ANOVA	Interaction	17 ; 729	F = 1.73	0.0329
		Pyk2f/f ; D1::Cre	22	22					Time	17 ; 729	F = 24.04	< 0.0001
									Genotype	1 ; 729	F = 35.32	< 0.0001
5B	Distance traveled (m/5 min)	Pyk2f/f	24	24				Two-way ANOVA	Interaction	17 ; 729	F = 2.381	0.0014
		Pyk2f/f ; D1::Cre	22	22					Time	17 ; 729	F = 52.38	< 0.0001
									Genotype	1 ; 729	F = 47.46	< 0.0001
5C	Distance traveled (m/15 min)	1st injection - Pyk2f/f	25	25		117	10	Two-way ANOVA Repeated measures	Interaction	1 ; 46	F = 2.65	0.11
		2nd injection - Pyk2f/f	25	25		168	9		Genotype	1 ; 46	F = 5.90	0.0191
		2nd injection - Pyk2f/f D1::Cre	23	23		102	8		Injection	1 ; 46	F = 42.60	< 0.0001
		1st injection - Pyk2f/f D1::Cre	23	23		132	7		Subjects (matching)	46 ; 46	F = 2.86	0.0003
								Sidak's multiple comparisons test	Pyk2f/f 1st inj vs 2nd inj	46	t = 5.89	< 0.0001
								Sidak's multiple comparisons test	Pyk2f/f ; D1::Cre 1st inj vs 2nd inj	46	t = 3.39	0.0029
5D	Locomotor response ratio, Day 14 / Day 1	Pyk2f/f	24	24	Yes			Mann Whitney's test two-tailed	Pyk2f/f vs Pyk2f/f ; D1::Cre		U = 228	0.44
		Pyk2f/f ; D1::Cre	22	22	No							
5E	Distance traveled (m/5 min)	Pyk2f/f	14	14		160	14	Two-way ANOVA	Interaction	17 ; 432	F = 0.17	> 0.99
		Pyk2f/f ; A2A::Cre	12	12		143	11		Time	17 ; 432	F = 14.73	< 0.0001
									Genotype	1 ; 432	F = 0.44	0.51
5F	Distance traveled (m/5 min)	Pyk2f/f	14	14				Two-way ANOVA	Interaction	17 ; 432	F = 0.22	0.99
		Pyk2f/f ; A2A::Cre	12	12					Time	17 ; 432	F = 38.01	< 0.0001
									Genotype	1 ; 432	F = 2.84	0.092
5G	Distance traveled (m/15 min)	1st injection - Pyk2f/f	14	14		95	10	Two-way ANOVA Repeated measures	Interaction	1 ; 24	F = 0.43	0.52
		2nd injection - Pyk2f/f	14	14		146	12		Genotype	1 ; 24	F = 0.014	0.91
		2nd injection - Pyk2f/f A2a::Cre	12	12		98	16		Injection	1 ; 24	F = 38.68	< 0.0001
		1st injection - Pyk2f/f A2a::Cre	12	12		139	13		Subjects (matching)	24 ; 24	F = 5.014	< 0.0001
								Sidak's multiple comparisons test	Pyk2f/f 1st inj vs 2nd inj	24	t = 5.06	< 0.0001
								Sidak's multiple comparisons test	Pyk2f/f ; A2a::Cre 1st inj vs 2nd inj	24	t = 3.79	0.0018
5H	Locomotor response ratio, Day 14 / Day 1	Coc Pyk2f/f	14	14	Yes	190	38	Mann Whitney's test two-tailed	Pyk2f/f vs Pyk2f/f ; A2A::Cre		U = 79	0.81
		Coc Pyk2f/f ; A2A::Cre	12	12	Yes	185	31					

Figure 6

Figure	Variable	Group	n mice	n data points	Failed normality test	Mean	SEM	Statistical analysis	Comparison	DF	Test value	*P* value
6A	Distance traveled (m/5 min)	SKF Pyk2f/f	20	20				Two-way ANOVA	Interaction	17 ; 801	F = 0.71	0.80
		SKF Pyk2f/f ; D1::Cre	27	27					Time	17 ; 801	F = 9.14	**< 0.0001**
									Genotype	1 ; 801	F = 5.80	**0.016**
6B	Distance traveled (m/5 min)	SKF Pyk2f/f	20	20				Two-way ANOVA	Interaction	17 ; 752	F = 0.47	0.96
		SKF Pyk2f/f ; D1::Cre	24	24					Time	17 ; 752	F = 26.32	**< 0.0001**
									Genotype	1 ; 752	F = 0.36	0.55
6C	Grooming	35-40 min	12	12	No	16	4	Student's t-test two-tailed unpaired	35-40 min vs 40-45 min	11	t = 2.91	**0.014**
		40-45 min	12	12	No	29	6					
6D	Distance traveled (m/45 min)	SKF 1st injection - Pyk2f/f	20	20		60,5	1,8	Two-way ANOVA Repeated measures	Interaction	1 ; 42	F = 2.23	0.14
		SKF 2nd injection - Pyk2f/f	20	20		75,0	2,8		Genotype	1 ; 42	F = 0.19	0.67
		SKF 1st injection - Pyk2f/f ; D1::Cre	24	24		56,4	1,9		Injection	1 ; 42	F = 83.86	**< 0.0001**
		SKF 2nd injection - Pyk2f/f ; D1::Cre	24	24		76,5	3,1		Subjects (matching)	42 ; 42	F = 2.51	**0.02**
								Sidak's multiple comparisons test	Pyk2f/f : 1st inj vs 2nd inj	42	t = 5.19	**< 0.0001**
								Sidak's multiple comparisons test	Pyk2f/f ; D1::Cre : 1st inj vs 2nd inj	42	t = 7.90	**< 0.0001**
6E	Locomotor response ratio, Day 14 / Day 1	SKF Pyk2f/f	20	20	No	1,3	0,05	Student's t-test two-tailed unpaired	Pyk2f/f vs Pyk2f/f ; D1::Cre	42	t = 1.61	0.11
		SKF Pyk2f/f ; D1::Cre	24	24	No	1,4	0,04					
6F	Distance traveled (m/5 min)	THX Pyk2f/f	13	13				Two-way ANOVA	Interaction	17 ; 414	F = 0.95	0.51
		THX Pyk2f/f ; D1::Cre	12	12					Time	17 ; 414	F = 19.11	**< 0.0001**
									Genotype	1 ; 414	F = 0.004	0.95
6G	Distance traveled (m/5 min)	THX Pyk2f/f	12	12				Two-way ANOVA	Interaction	17 ; 396	F = 0.60	0.89
		THX Pyk2f/f ; D1::Cre	12	12					Time	17 ; 396	F = 20.46	**< 0.0001**
									Genotype	1 ; 396	F = 0.44	0.51

Figure 7

Figure	Variable	Group	n mice	n data points	Failed normality test	Mean	SEM	Statistical analysis	Comparison	DF	Test value	*P* value
7A	Time spent in the cocaine-paired arm (sec)	Day 0 Pyk2+/+	10	10		402	26	Two-way ANOVA Repeated measures	Interaction	1 ; 17	F = 0.036	0.85
		Day 7 Pyk2+/+	10	10		557	28		Genotype	1 ; 17	F = 1.90	0.19
		Day 0 Pyk2-/-	9	9		374	30		Injection	1 ; 17	F = 17.90	**0.0006**
		Day 7 Pyk2-/-	9	9		515	37		Subjects (matching)	17 ; 17	F = 0.52	0.90
								Sidak's multiple comparisons test	Pyk2+/+: Day 0 vs Day 7	17	t = 3.21	**0.01**
								Sidak's multiple comparisons test	Pyk2-/-: Day 0 vs Day 7	17	t = 2.79	**0.0252**
7B	CPP score (s)	Pyk2+/+	10	10	No	154	46	Mann Whitney's test two-tailed	Pyk2+/+ vs Pyk2-/-		U = 39	0.64
		Pyk2-/-	9	9	No	141	53					
7C	CPP score (s)	Pyk2f/f ; NAc GFP	18	18	No	114	30	Mann Whitney's test two-tailed	Pyk2f/f ; NAc GFP vs NAc Cre, GFP		U = 112	0.41
		Pyk2f/f ; NAc Cre, GFP	15	15	No	83	32					
7D	CPP score (s)	Pyk2f/f ; DS GFP	8	8	No	142	47	Mann Whitney's test two-tailed	Pyk2f/f ; DS GFP vs DS Cre, GFP		U = 27	0.96
		Pyk2f/f ; DS Cre, GFP	7	7	Yes	146	42					
7E	CPP score (s)	Pyk2f/f	13	13	No	135	40	Mann Whitney's test two-tailed	Pyk2f/f vs Pyk2f/f ; D1::Cre		U = 60	0.34
		Pyk2f/f ; D1::Cre	12	12	No	160	21					
7F	CPP score (s)	Pyk2f/f	14	14	No	170	26	Mann Whitney's test two-tailed	Pyk2f/f vs Pyk2f/f ; A2a::Cre		U = 69	0.45
		Pyk2f/f ; A2a::Cre	12	12	No	137	35					

Figure 8

Figure	Variable	Group	n mice	n data points	Mean	SEM	Statistical analysis	Comparison	DF	Test value	*P* value
8B	pGluN2B (% of Pyk2f/f saline)	Pyk2f/f , saline	10	10	100	7	Two-way ANOVA	Interaction	1 ; 42	F = 1.36	0.86
		Pyk2f/f , cocaine	11	11	124	4		Genotype	1 ; 42	F = 0.17	0.19
		Pyk2f/f ; D1::Cre , saline	13	13	104	3		Drug	1 ; 42	F = 10.89	**0.002**
		Pyk2f/f ; D1::Cre , cocaine	12	12	115	7					
							Sidak's multiple comparisons test	Pyk2f/f saline vs cocaine	17	t = 3.03	**0.008**
							Sidak's multiple comparisons test	Pyk2f/f ; D1::Cre saline vs cocaine	17	t = 1.58	1.58
8B	Total GluN2B (% of Pyk2f/f saline)	Pyk2f/f , saline	10	10	100	6	Two-way ANOVA	Interaction	1 ; 41	F = 1.36	0.67
		Pyk2f/f , cocaine	11	11	106	5		Genotype	1 ; 41	F = 0.17	0.62
		Pyk2f/f ; D1::Cre , saline	12	12	100	3		Drug	1 ; 41	F = 10.89	0.49
		Pyk2f/f ; D1::Cre , cocaine	12	12	101	6					
8C	pERK (% of Pyk2f/f saline)	Pyk2f/f , saline	5	5	100	4	Two-way ANOVA	Interaction	1 ; 19	F = 0.07	0.79
		Pyk2f/f , cocaine	6	6	131	10		Genotype	1 ; 19	F = 0.70	0.41
		Pyk2f/f ; D1::Cre , saline	6	6	109	8		Drug	1 ; 19	F = 13.69	**0.0015**
		Pyk2f/f ; D1::Cre , cocaine	6	6	136	8					
							Sidak's multiple comparisons test	Pyk2f/f saline vs cocaine	17	t = 2.74	**0.026**
							Sidak's multiple comparisons test	Pyk2f/f ; D1::Cre saline vs cocaine	17	t = 2.49	**0.044**
8C	Total ERK (% of Pyk2f/f saline)	Pyk2f/f , saline	5	5	100	4	Two-way ANOVA	Interaction	1 ; 19	F = 0.06	0.80
		Pyk2f/f , cocaine	6	6	102	5		Genotype	1 ; 19	F = 0.03	0.85
		Pyk2f/f ; D1::Cre , saline	6	6	100	5		Drug	1 ; 19	F = 0.01	0.92
		Pyk2f/f ; D1::Cre , cocaine	6	6	100	7					

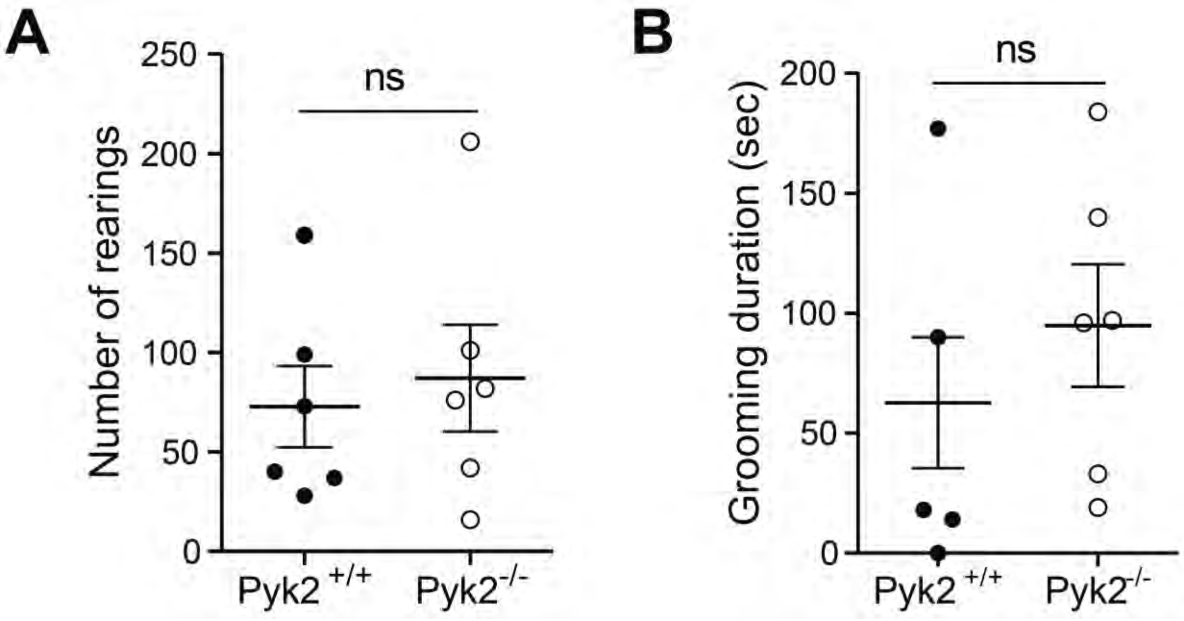

Supplementary Figure 1. Effects of cocaine on stereotyped behavior in Pyk2$^{+/+}$ and Pyk2$^{-/-}$ mice. The number of rearings (**A**) and the time spent self-grooming (**B**) were quantified during the 40 minutes after the 1st cocaine injection in 6 Pyk2$^{+/+}$ and 6 Pyk2$^{-/-}$ mice randomly selected from those shown in **Fig. 3C**. Means ± SEM are indicated by horizontal bars. Statistical analysis was done with two-tailed Mann and Whitney's test. Pyk2$^{+/+}$ vs Pyk2-/-, number of rearings, $U_{6,6} = 14$, $p = 0.59$, duration of grooming, $U_{6,6} = 9$, $p = 0.18$. n.s, not significant.

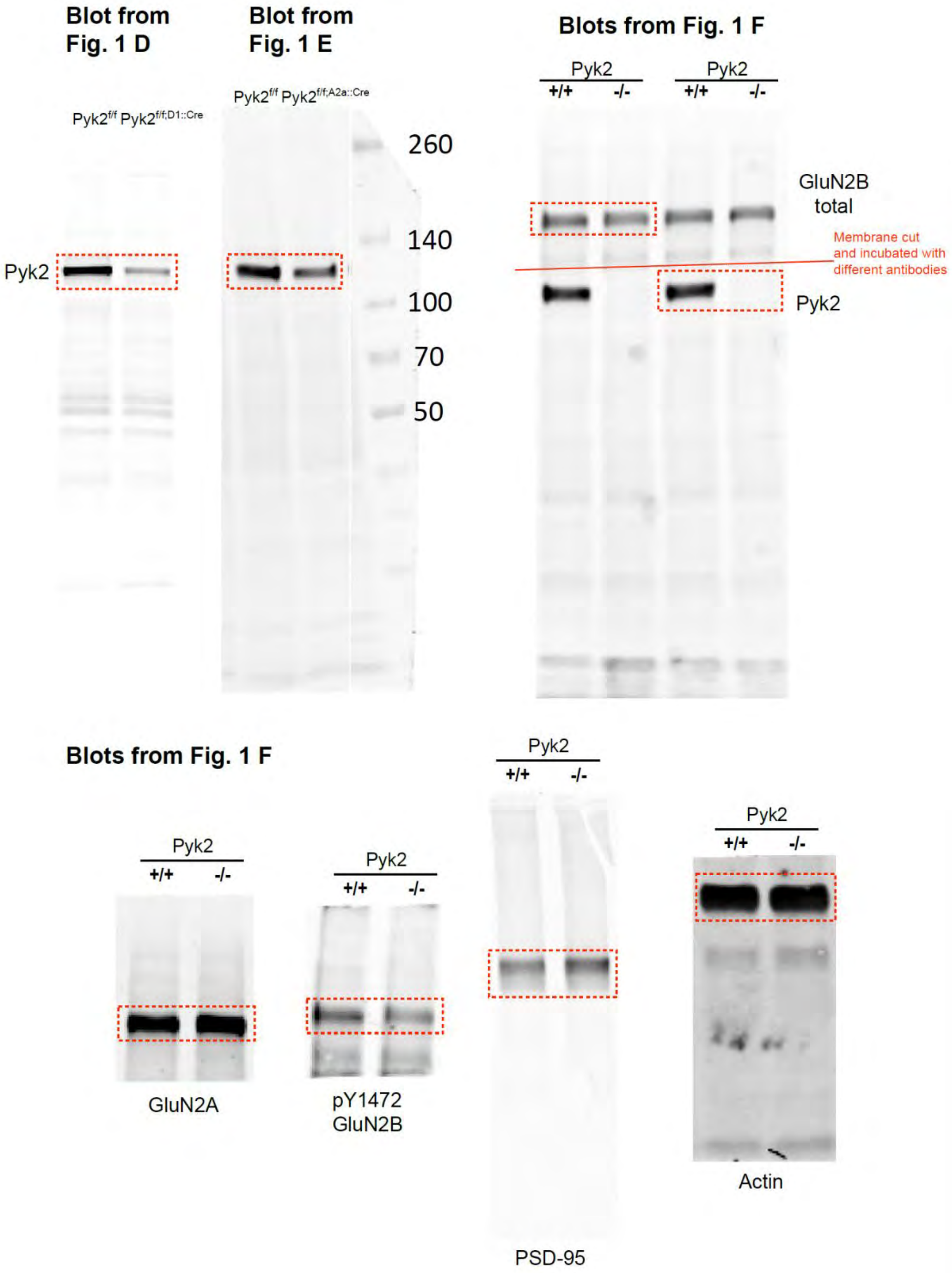

Supplementary Figure 2: Full length blots from Fig. 1D, E, and F.

Blots from Fig. 1 H

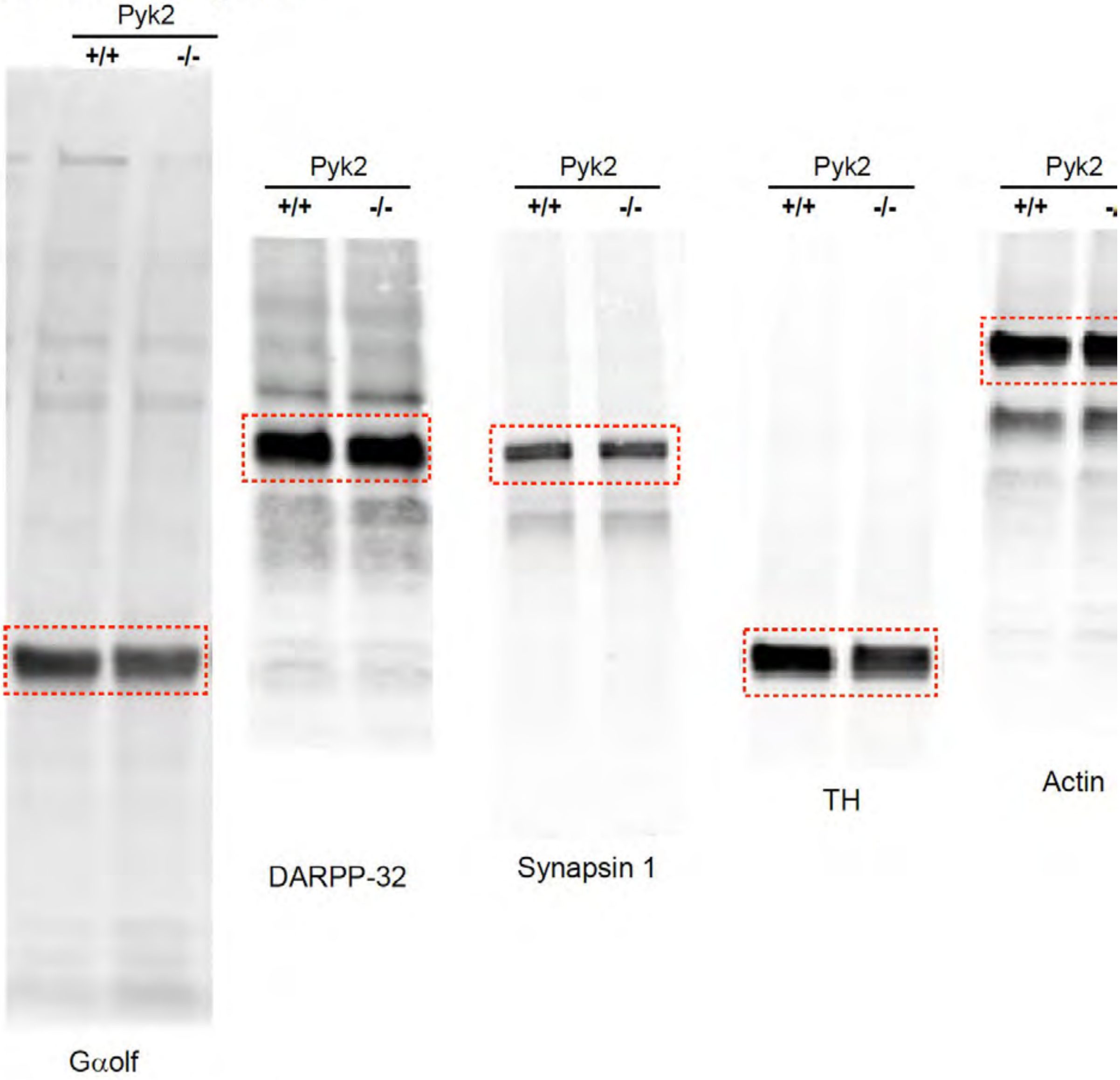

Blots from Fig. 3A

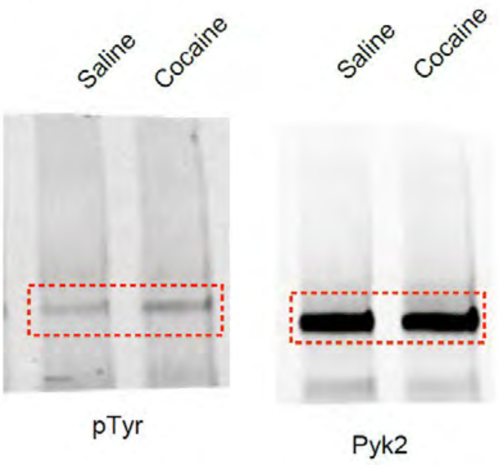

Supplementary Figure 3: Full length blots from Fig. 1H and 3A.

Blots from Fig. 8A

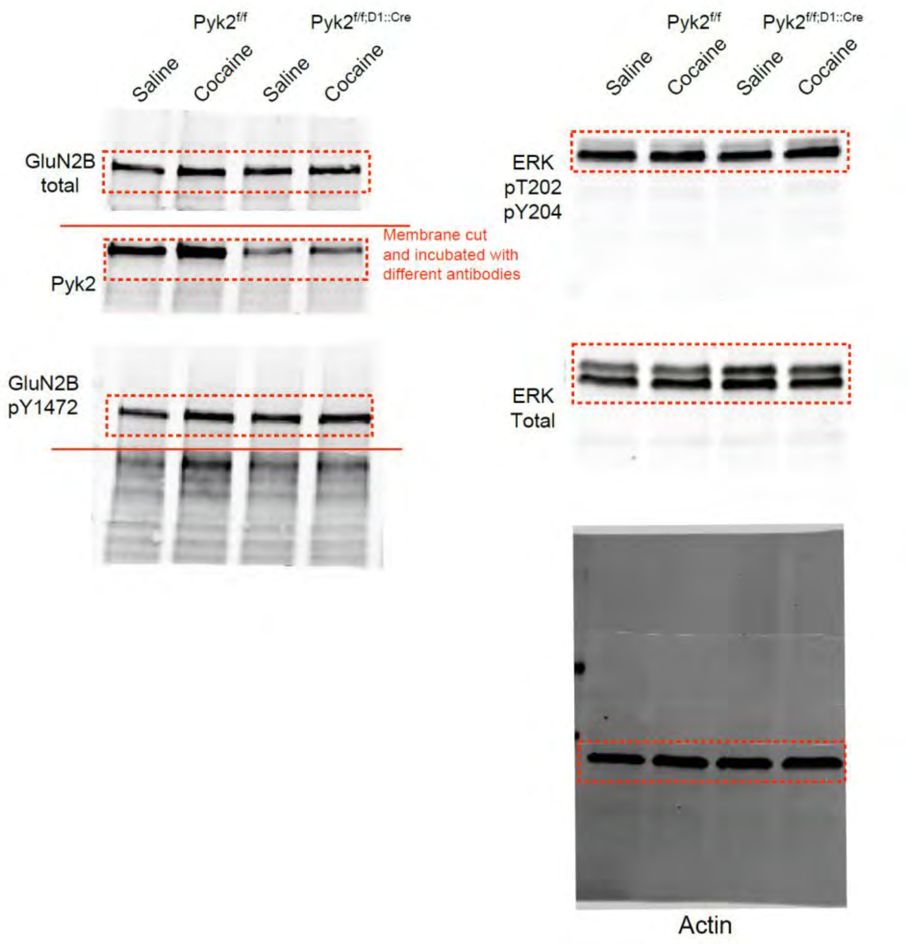

Supplementary Figure 4: Full length blots from Fig. 8A.
